# Physical properties of odorants affect behavior of trained detection dogs during close-quarters searches

**DOI:** 10.1038/s41598-024-55323-y

**Published:** 2024-02-28

**Authors:** Daniel Mejia, Lydia Burnett, Nicholas Hebdon, Peter Stevens, Alexis Shiber, Clay Cranston, Lauryn DeGreeff, Lindsay D. Waldrop

**Affiliations:** 1 Schmid College of Science and Technology, Chapman University, Orange, CA 92866 USA; 2 Global Forensic and Justice Center and Department of Chemistry and Biochemistry, Florida International University, Miami, FL 33199 USA; 3 The Scentsable K9, El Cajon, CA 92021 USA

**Keywords:** Chemical ecology, Animal behaviour

## Abstract

Trained detection dogs have a unique ability to find the sources of target odors in complex fluid environments. How dogs derive information about the source of an odor from an odor plume comprised of odorants with different physical properties, such as diffusivity, is currently unknown. Two volatile chemicals associated with explosive detection, ammonia (NH_3_, derived from ammonium nitrate-based explosives) and 2-ethyl-1-hexanol (2E1H, associated with composition C4 plastic explosives) were used to ascertain the effects of the physical properties of odorants on the search behavior and motion of trained dogs. NH_3_ has a diffusivity 3.6 times that of 2E1H. Fourteen civilian detection dogs were recruited to train on each target odorant using controlled odor mimic permeation systems as training aids over 6 weeks and then tested in a controlled-environment search trial where behavior, motion, and search success were analyzed. Our results indicate the target-odorant influences search motion and time spent in the stages of searching, with dogs spending more time in larger areas while localizing NH_3_. This aligns with the greater diffusivity of NH_3_ driving diffusion-dominated odor transport when dogs are close to the odor source in contrast to the advection-driven transport of 2E1H at the same distances.

## Introduction

Dogs are trained to locate items of interest via the detection of chemical cues (odors), including contraband, firearms, explosives, disease, and humans^1–7^. Trained operational dogs are a key feature of many security and law enforcement programs, despite the cost and difficulty of selecting, training, and maintaining them^8,9^. Technology of artificial sensors now rivals the sensitivity of a dog’s nose, yet dogs continue to out perform these sensors in their superior selectivity and their ability to interpret the complex fluid problem of locating a target odor’s source^1–3,10,11^.

Dogs must search a spatially and temporally complex odor plume that is created by the interactions between an odor source and the environment^2,12,13^. Odor enters the surrounding air from the source and is carried away via air currents^13,14^. Air shearing across itself forms high-concentration filaments of odor in eddies, and molecular diffusion spreads odors between layers of air in eddies, softening the edges of the odor filaments^13,15^. This mixing is incomplete, and as a result, an odor plume consists of patterns of high- and low-concentration filaments that vary in space and time^15^. Both environmental features, such as wind and objects in the area, and the physical properties of an odorant, such as diffusivity, can impact the distribution and concentration of the odor filaments within the plume^13,16^.

When a dog investigates a plume, it samples the plume by sniffing, or taking in discrete volumes of the air immediately surrounding its nose^2,17^. The signals that the dog encounters at the scale of its nose are intermittent; odor is contained in high-concentration filaments which the dog samples between low- or no-odor samples^11,15^. Thus, odor information from a plume is complex in space and time and not a steady concentration gradient from start to source^2,12,13^.

**Table 1:** Estimated Péclet numbers (*Pe*) for different length scales where the flow speed (*U*) is 1 cm s^-1^ and the diffusion coefficients (*D*) are 0.0663 (2E1H), 0.2403 (NH_3_), 0.0591 (bromooctane), and 0.0711 (methyl benzoate) cm^2^ s^-1^.

	Length, *L* (m)	*Pe* 2E1H	*Pe* NH_3_	*Pe* bromooctane	*Pe* methyl benzoate
Far field	10.0	754	208	8460	7032
Medium range	1.0	75	21	846	703
Close quarters	0.1	8	2	85	70

Through this sampling, dogs must derive enough information from a plume to find the source of an odor. Dogs and other animals use two sets of behaviors during a search to gather information from an intermittent odor plume: casting and localizing^2,18^. Casting is the appearance of zig-zagging in moths and crabs, constant turning in fish and birds, or head turning in mice and dogs^2,12,18–22^. Casting typically occurs far from the source and allows the animal to enter, exit, and re-enter the edges of the plume, acquiring information on the odor’s source from the spatial and temporal patterns of signals as well as flow direction and speed^18–20,23,24^. Once very close to the source, these signals become less reliable and animals switch to localizing behaviors to determine the exact position of the odor source^11,18,25,26^.

The shape of the plume depends on advection and diffusion, which can influence the way odor moves local to the source. Whereas vapor pressure of odorants largely determines the mass of that odorant that enters the surrounding air, molecular diffusion determines how much that mass spreads out in the air. The Péclet number describes the ratio between advection and diffusion in odor transport: $Pe = UL/D$, where *U* is the external flow speed, *L* is a characteristic length, and *D* is the diffusion coefficient of the odorant. When considering transport of odor far from the source (*L* is large), the *Pe* is much greater than one, indicating that bulk air movement is the primary mode of transport and differences in diffusivity have little importance. However, when considering close-quarters searching, characteristic lengths would be much smaller. Here, even moderate differences in diffusivity between odorants can drive *Pe* close to one, indicating the relative importance of diffusion-driven transport (Table 1).

We know that environmental processes that affect advection can produce effects in search behavior and success in dogs that engage in long-distance searching^3,27,28^, but there have been fewer studies investigating how an odor’s physical properties may impact close-quarters searches. Many operational dogs engage in close-quarters searching for contraband and explosives in rooms or enclosed spaces or items, such as vehicles, packages, or luggage, where the dog begins its search close to the odor source. While odors of different physical properties can also affect search success of narcotics and explosive detection dogs^1^, there has been no study on how these differences might affect the behavior and movement of dogs during the casting and localizing stages of active searches. Since these types of searches can have $Pe \approx 1$, it is possible that odors of different diffusivities could impact the way dogs search and their success in close quarters.

**Figure 1:**
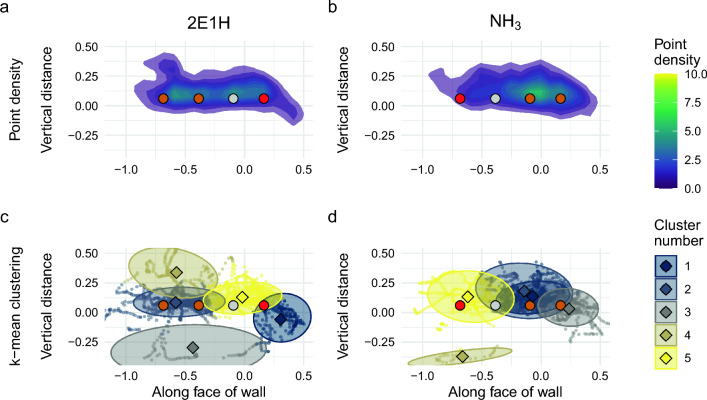
Point-density plots (**a**,**b**) and k-means clustering plots (**c**,**d**) of the front view of trial wall during casting stage for 2E1H (**a**,**c**) and NH_3_ (**b**,**d**). Location of imprinting source holes marked with colored circles (red indicates location of target odorant, dark orange are distractors, gray is blank).

To investigate how the physical properties of odors can impact close-quarters searching and search success, we observed the unrestricted search behavior of trained dogs in a standardized search environment with one of two target odorants: ammonia (NH_3_) and 2-ethyl-1-hexanol (2E1H). These chemicals are important for detection of explosives, as they are the dominant odorants in the headspace of two common explosives (NH_3_ is a degradant of ammonium nitrate and 2E1H is associated with the plasticizers used in the plastic explosive, C4)^1,29–33^. NH_3_’s diffusion coefficient is 3.6 times that of 2E1H (Table 1)^34–36^.

We expected the behavior and motion of the dogs would differ between the two target odorants due to their physical properties, with movement distributed more widely during localizing NH_3_, where $Pe \approx 1$. We also predicted that behaviors associated with stress, such as lip licking, as well as more spread movements would occurring searches for NH_3_ than 2E1H due to the much wider, more diffuse plume produced by NH_3_.

## Results

### Target odor affects search patterns of dogs

**Figure 2:**
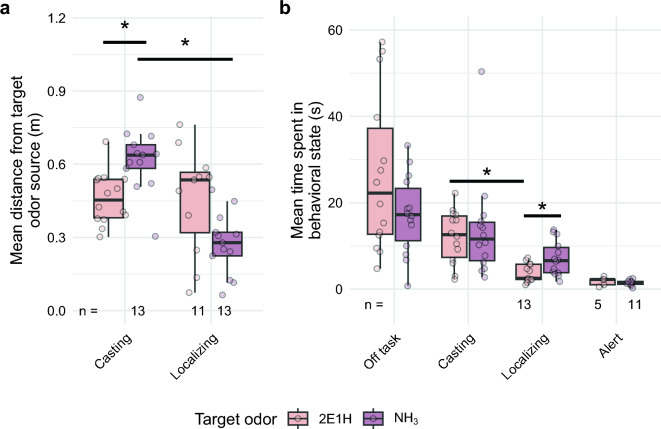
Mean distance the dogs occupied in the casting and localizing stages of search (**a**) as well as the mean time they spent within 10 cm of the source of each chemical (**b**). Single black filled circles represent outliers to the box plot. Sample size is noted along the bottom line if other than n = 14. * represents a comparison where the P < 0.05.

The casting stage describes the active search of a dog at the edge of a plume before it begins localizing to narrow down the search field to the odor source. It often involves larger movements and more changes of direction in order to cover a larger area and encounter infrequent odor signals. During casting, dogs approached the wall and worked between 0.2 and 1.0 m away from the wall. Figure 1 show the density maps of casting across all runs for both target odors. The distances of the dogs’ noses to the target odor source differed significantly (Fig. 2a, means: $0.619$ m and $0.461$ m for 2E1H and NH_3_, respectively; $t = -3.41$; $P = 0.0096$). The time spent engaging in casting, however, did not differ. Casting occurred further from the face of the wall and spread across the face of the wall (Fig. 1). K-mean gap scores for clusters were overall low for both 2E1H (0.63, 0.74, 0.71, 0.75, 0.76) and NH_3_ (0.75, 0.73, 0.71, 0.72, 0.76).

**Figure 3:**
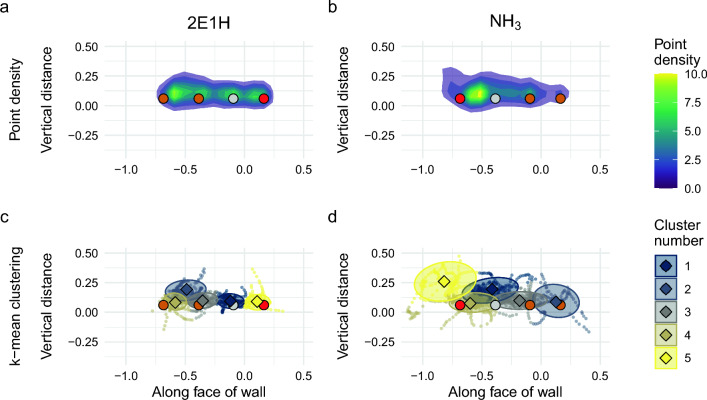
Point-density plots (**a**,**b**) and k-means clustering plots (**c**,**d**) of the front view of trial wall during localizing stage for 2E1H (**a**,**c**) and NH_3_ (**b**,**d**). Location of imprinting source holes marked with colored circles (red indicates location of target odorant, dark orange are distractors, gray is blank).

Localizing describes the behavior in which a dog searches a smaller area to narrow down the position of the odor source. This smaller search area is reflected in the localizing point density of 2E1H (Fig. 3a,b), with the searching highly localized to the holes of the wall and little deviation from the wall’s face. Clustering is highly robust and four of the groups correspond to the four source holes of the wall (Fig. 3c,d; gap scores of 0.58, 0.90, 0.91, 0.90, 0.90). In contrast, NH_3_ localizing, while close to the surface of the wall, was spread both above and below the holes, showing clusters that were less distinct and not associated with individual holes (gap scores: 0.64, 0.74, 0.74, 0.70, 0.75). Dogs were closer to the target source during localizing NH_3_ (Fig. 2a) compared to 2E1H (Welch Two Sample t-test; means: $0.275$ m and $0.455$ m away from the wall for NH_3_ and 2E1H, respectively; $t = 2.41$; $P = 0.12$). For NH_3_, the dogs had the highest density of movement near the odor source, whereas they searched the entire wall tightly for 2E1H (Fig. 3a,b). There was a slightly higher density on the opposite end of the wall from the 2E1H odor source, yet the dogs still searched more tightly around all the holes than for NH_3_ (Fig. 3a,b). Dogs also spent a longer time engaged in localizing for NH_3_ than 2E1H (Fig. 2b) (Welch Two Sample t-test; means: $7.09$ s, $3.72$ s, $P = 0.048$). With this, the time dogs engaged in casting as opposed to localizing NH_3_ did not differ (Wilcoxon rank sum exact test, $W = 143$, $P = 0.079$), yet the dogs spent significantly less time localizing 2E1H than casting for it (Welch Two Sample t-test; means: $3.72$ s, $12.2$ s, $P = 3\times 10^{-4}$).

### Dog behavior during search does not change with target odorant

**Figure 4:**
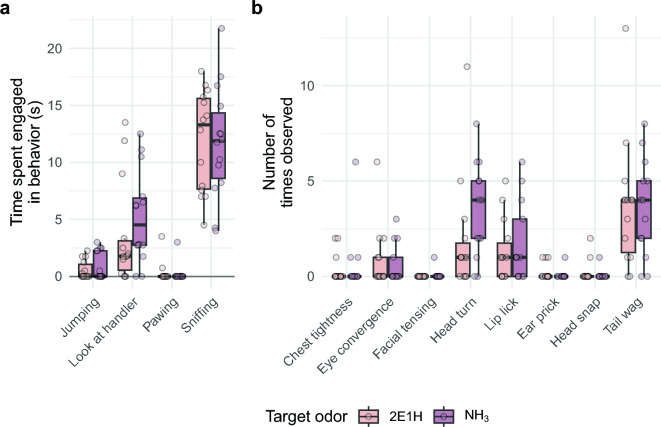
Mean time spent showing each of four behaviors (**a**) and the number of times the dogs displayed each of eight other behaviors (**b**). There were no significant difference in any pairwise comparisons.

When looking at both the counts and time spent displaying 12 behaviors associated with searching, focus, stress, and alerts, no significant differences were found between the two target odors. In Fig. 4a, the times the dogs spend displaying four state behaviors was not found to be statistically different based on the target odor (two-way ANOVA, DFs = $1, 3$; F-statistics = $0.52, 0.50$; P values = $0.47, 0.69$). No individual comparison between target odor within a specific state were significant. The dogs did display a slight trend towards a difference when looking at the handler based on the target odor. However, a small sample size limited the statistical power of this comparison.

Furthermore, the time spent sniffing for both target odors did not differ statistically (Welch’s Two Sample t-test; means: $11.7$ s, $11.9$ s, $P = 1$), therefore demonstrating that the dogs spent about equal amounts of time searching for the two odors.

The counts of individual behaviors, including those that signify states of being, also did not result in any significant differences in how the dogs searched for the target odors (Fig. 4b). While the number of times the dogs turned their heads towards their handlers seemed to trend towards occurring more for NH_3_, the result was also insignificant ($P = 0.37$). These results suggest there was no difference in states of being for dogs engaged in searching for either target odor.

### Search success changes with target odorant

**Figure 5:**
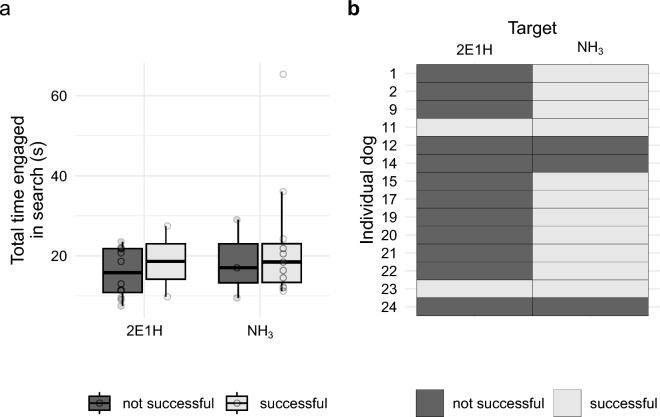
Mean search times between types of runs (**b**) against alert success for both chemicals (**a**). Each point represents the mean for a single dog search event b.

In the trial, dogs had five choices for each search: alerting on one of each imprinting hole or not alerting at all. During the 2E1H trial, alerts were recorded as 2/14 (target), 1/14 (blank), 1/14 (methyl benzoate distractor), 0/14 (bromooctane distractor), 10/14 (no choice). For NH_3_, alerts were recorded for 11/14 (target), 0/14 (blank), 0/14 (methyl benzoate distractor), 0/14 (bromooctane distractor), 3/14 (no choice). Unexpectedly, these results indicate that 2/14 dogs were successful at finding 2E1H and 11/14 were successful at finding NH_3_, a difference which is significant (Fisher’s exact test, $P = 0.002$; Fig. 5a).

With this search success difference, a difference in the total time engaged for each target odor between successful and unsuccessful searches was not found. The successful searches did not differ in time from the unsuccessful searches for both chemicals (Wilcoxon rank sum exact test, $W = 19$, $P = 0.769$ for NH_3_ and $W = 15$, $P = 0.66$ for 2E1H) or between the chemicals (Wilcoxon rank sum exact test, $W = 13$, $P = 0.77$ for successful searches and $W = 20$, $P = 0.84$ for unsuccessful searches; Fig. 5b).

## Discussion

The target odorants in this study possess differences in physical properties such that during close-quarters searching, transport of the odorant would be more dominated by diffusion for NH_3_ and advection for 2E1H during localizing (Table 1). We predicted that this difference would result in significant differences in the way dogs would search odor plumes created by each target odorant, displayed by differences in movement, behavior stages of the search, and individual behaviors associated with focus and distress. Results show that the dogs engaged in different patterns of movement during searches for 2E1H and NH_3_ without changing the rate of individual behaviors associated with focus and distress.

Together, these results suggest that the physical properties of NH_3_ and 2E1H influence the movement of the dogs during search. The differences in plume characteristics between the two target odorants in this study made it easier for dogs to identify the edges of the plume during casting, where both target odors have $Pe>> 1$ in the medium range (Table 1). But the movement patterns of dogs diverged considerably where NH_3_ has $Pe \approx 1$ and 2E1H has $Pe > 1$. The more spread, diffuse plume of NH_3_ required more time during localizing. Anecdotally, many handler participants reported that their dogs seemed to react differently in movement and search patterning during training with NH_3_ as compared to training with 2E1H. NH_3_ seemed to present a novel challenge for the dogs. The differences in properties also required the dogs to search in different areas with different movements in response to plume characteristics between NH_3_ and 2E1H. Other studies investigating odor capture by crabs across this *Pe* value between air and water found support for morphological and kinematic differences being a result of performance differences in the two fluid environments^37,38^. This transition corresponds roughly to the behavioral transition between casting and localizing, observed by many studies that show distinct changes in behavior when an animal is so close to the source that the intermittent cues of a plume become less reliable^18,24,25^. Our results are consistent with these observations and suggest that the target odorant’s diffusivity can drive where and when this transition occurs for dogs during a search.

Although the movements of dogs during the searches differed, individual behaviors did not (Fig. 4). Results indicated that dogs engaged in the same set of behaviors for each odorant, maintaining similar levels of focus and distress. This implies that the dogs were neither averse to the target odorants nor experienced different levels of distress during the trial searches. There was a slight, but insignificant, trend toward dogs looking at their handlers during the NH_3_ search unrelated to alert trained final responses, which may have reached significance with a larger sample size.

We predicted that the difference in physical properties between the two target odorants would lead to dogs having more success at finding 2E1H as it is more similar in physical properties to the nose work odors that the dogs had been previously trained to locate. Nose work odors, the essential oils of birch, anise, and clove, consist broadly of turpenes which have more similar vapor pressures and diffusivities to 2E1H as compared to NH_3_. However, dogs successfully alerted on NH_3_ significantly more often than 2E1H. We attribute greater success in alerting to NH_3_ to the greater plume size of NH_3_ compared to 2E1H, making more odor available further outside the imprinting hole. While the COMPS devices delivering odor matched the mass per unit time released into the air, greater diffusivity would result in more NH_3_ escaping the imprinting enclosure via the small hole on the front panel (see SI Fig. 1).

The interesting observation of difference in search success between the two target odors raises a potential limitation of the study. There are two explanations for these results: (1) the dogs were not able to recognize the target odor because they did not pick up enough odor to localize the source, or (2) the dogs were not able to recognize the target odor because they were not sufficiently trained to do so. While our experiment was not designed to, and is ultimately unable to, distinguish between differential success rates, it was an interesting result. There is evidence that suggest that dogs were sufficiently trained and not able to pick up enough 2E1H to localize the source. Dogs trained the same amount of time on the same aides as NH_3_ with high success rates for NH_3_. Dogs showed distinct kinematic and behavior differences after training. Before training, when the target odor and all distractors were novel, dogs spent less time localizing and showed spread kinematic densities during localizing for both target odors (NH_3_ and 2E1H) (SI Fig. 4a,b). After training, dogs spent more time localizing, showing different movement patterns between target odors (SI Fig. 4c,d) which indicates they were engaged in the search task and detected enough 2E1H during the post-training trial to transition from casting to localizing more often than in the pre-training trial. Furthermore, dogs also showed proficiency before and after the target trial using a similar device with nose work essential oils.

Few studies have investigated kinematic or behavioral changes of animals engaged in searching for odorants of different physical properties. Most odors are a mixture of individual chemical odorants, each with their own physical properties^25^. Many common explosives give off multiple chemical odorants, and when trained dogs are challenged with odorants individually, they are less proficient in detection^39,40^. Each odorant has individual physical properties, such as diffusivity, which can influence the shape of the plume or the spatial and temporal pattern of signals delivered to dogs^41–43^. It is possible that dogs use not only the spatial and temporal information from each odorant as it is transported in the plume, but also the changing chemical makeup of the odor that they capture during different stages of the search.

Understanding that a dog’s movements during a search will be different, while individual behaviors may not, is important for the handler when reading their dog’s behavior during a search. Trained final responses are not the only signals handlers should learn to discriminate, the specific behaviors and movements of dogs during the search can be nuanced and indicate the stage of the search activity, the possible locations of sources, and possibly, the type of odors being searched for. A better understanding of differences in the search process can improve a handler’s ability to intervene and guide the dog, communicate with other people, improve reliability, and maintain safety in a dangerous working environment^4,29,44–47^.

This novel exploration into the detailed ways that dogs search for odors based on the physical properties of odorants can also be useful for the wider investigation into and creation of artificial sensors. Beyond simply the sensitivity of the sensor itself, search patterning is a necessary component for locating various targets. Search patterns, such as the zig-zag and spiral search patterns, as well as other strategies have been taken successfully from animal models for use in robotics work^48–51^. Study of the detailed methods in which animals such as dogs search for odor plumes of different target odors will aid in the growth of artificial sensors and their ability to discriminate and locate specific odors.

Fourteen dogs participated and, while this sample size was adequate and gathered telling data, it may have also served as a limitation, as evidenced by a trend and non-significant result for more time spent looking at the handler when searching for NH_3_. A larger sample size may have provided more statistically significant results for the study of individual behaviors and for alert success. With this, the dogs were only trained for 6 weeks on the target chemicals, and while there was a clear difference in their ability to and in how they searched for the odors, these differences may have been affected by the length and presence of training. Moreover, operational dogs were not utilized, instead, nose work dogs registered through the National Association of Canine Scent Work^TM^ participated. This may have been a limitation as there may be training, behavioral, or search-pattern differences between operational and non-operational dogs. Yet the use of non-operational dogs is consistent with the participant population of the wider literature^21,44,52^. These limitations serve as a call for continued exploration on the subject to propel beneficial developments of canine searching for all operational capacities.

## Methods

### Materials

Ammonium hydroxide (Sigma-Aldrich) and 2-etyhyl-1-hexanol (Sigma-Aldrich) were selected as the two target odorants of interest due to differences in their physical properties (Table 1; SI Table 1). In order to train the dogs to search the two different target odors, handlers were given controlled odor mimic permeation systems (COMPS) as training aids. COMPS are a tool that controls odor availability during training. For this study, each COMPS consists of 20 $\upmu$L of analyte pipetted onto a 2 in. by 2 in. piece of cotton-blend gauze pad (Dukal, LLC) which was heat-sealed in a 2 in. by 3 in. plastic bag. The analyte is released at a rate determined by the thickness and surface area of the bag^53^. Since NH_3_ has a much higher vapor pressure than 2E1H, it was necessary to equalize the amount of each chemical presented to the dog during training and the experimental trial. In order to accomplish this, a 3 MIL low density polyethylene bag (LDPE; Uline) was used for 2E1H and a 8 MIL LDPE bag (Uline) was used for NH_3_. Non-target or distractor odorants were prepared in a similar way for methyl benzoate (Sigma-Aldrich; 3 MIL bag) and bromooctane (Sigma-Aldrich; 8 MIL bag).

### Participants

Fourteen civilian dogs were recruited through the National Association of Canine Scent Work (NACSW^TM^). NACSW dogs train to detect essential oils for competition. Dogs recruited had achieved NACSW Nose Work 3 title or higher. Dogs at this level are trained to detect multiple odor sources, trials with blanks, and in various search situations. Three small dogs (dogs $< 30$ lbs; mean body weight 9.2 lbs) and eleven large dogs (dogs $\ge 30$ lbs; mean body weight 55.7 lbs) were included of various breeds (SI Table 2). The handlers were not compensated for participation. During the training period, handlers were given COMPS training aids containing either 2E1H, NH_3_, or a blank aid with gauze sealed in a bag and no analyte. Trainers logged the time in which they used each COMPS and were instructed to throw away the COMPS and open a new one after it was exposed to the air for a total of 6 h. Handler-dog teams were given 6 weeks to train with the COMPS before participating in the trial. Each handler-dog team trained off-site and individually as they would for typical nose work odor competitions.

### Experimental setup and procedure

The trial was held in Huntington Beach, California in February 2023 at a dog training facility. Two plywood trial walls were set up in the middle of facility (SI Fig. 1). The walls consisted of three four-foot-by-four-foot panels of half-inch thick plywood with 2 in.-by-3-in. studs that were connected in a C pattern. The center panel contained four imprinting holes (3-in. interior, 4-in. exterior ABS toilet flange; OATEY) located at a distance on center from the bottom of the wall. In each of the four flanges, a 3-in. white cap was secured in each opening, holding a sensor. Each hole appeared identical to both the dogs and human observers. Two experimental walls were used corresponding to the size of the dogs in the study with the only difference being the distance of the imprinting holes from the bottom of the wall: 16 in. (0.406 m) for large dogs and 10 in. (0.254 m) for small dogs (SI Fig. 1). Each hole presented a single odorant to the dogs through a small opening on a plastic PVC cap (SI Fig. 1). Behind the wall and not visible to the dogs, the interior side of the flanges each had a white PVC pipe extending horizontally from the face of the wall. One COMPS was placed in three of the four holes, containing one of two distracting odorants (either methyl benzoate or bromooctane) or one of the two target odorants (either 2E1H or NH_3_). A fourth hole had no odor (blank). For additional details, see supplementary information.

COMPS were placed in the imprinting devices 5–10 min before dogs were allowed to interact with the wall. The dogs were then run in no specific order. Once one dog left the experimental area, another dog-handler dyad was brought into the area. No special effort was made to reset or control the odor plume from the wall and the area was not cleaned between dogs. All dogs were first run with 2E1H and then NH_3_. After each run with a target odor, distracting-odor COMPS were replaced. The white, PVC cap and ABS imprinting hole were replaced between 2E1H and NH_3_ runs to limit cross-contamination between target odors. A 15- to 20-min period between runs with each target odor was given to allow the previous odor plumes to dissipate.

During a run, each dog was allowed to first search a similar imprinting wall with a nose work odor source immediately before each experimental run. Two observers remained behind the dog-handler dyad to note observations and to coach the handler, if needed. The handlers were blind to which hole contained the target odor. Dogs were allowed to freely move about the face and sides of the wall during the search while remaining on a long leash. Dogs then approached the wall to search until either a trained final response was called by the handler or the handler determined the dog was not likely to find the target source. Once the dog had searched the experimental wall, the dog was given a second opportunity to search the wall containing a nose work odor source and then the team exited the facility. To assess whether the dogs were more successful at locating one odor or another, the successful versus unsuccessful alerts were noted by both observers during each run.

### Kinematic analysis

Two video cameras (GoPro Hero 8 Black, GoPro, San Mateo, CA) were placed in view of the front of the wall where dogs interacted with odors, one above the holes and one to the right side of the trial wall, resulting in a top view and side view, respectively (SI Fig. 2). Calibration footage was taken each time the cameras were initially set up or moved. For additional details, see supplementary information. Movements of dogs during the search were quantified using the tracking software DLTdv8^54^. Two points on the dogs’ heads (the middle of the tip of the nose, in between the dogs’ eyes) were digitized by hand in each frame of the two synced video views in DLTdv8. Ten calibration points were also digitized per run. The two dimensional points from each video view were then used with easyWand calibration to calculate the three-dimensional position of each digitized point^55^. Three-dimensional points were then exported for further analysis using custom software in R version 4.3.1^56^ (see data availability for location of code used for analyses). The three-dimensional kinematic points were then realigned with the trial wall’s face through the use of calibration points such that the bottom center of the ABS imprinting holes represented 0 on the vertical axis, aligning the kinematic track points from the small and large dog walls. The same two coders for the behavioral analysis completed the kinematic analysis, with one coder acting as a confirmatory judge of the first coder’s analysis.

### Behavioral analysis

To analyze the behaviors occurring along the search path, two ethograms were created to code behavioral stages related to searching and other, relevant individual behaviors that occurred during the search. Each ethogram was created and coded using the Behavioral Observation Research Interactive Software (BORIS)^57^. Behavior states and individual behaviors were scored by two trained observers (one for behavior states, one for individual behaviors).

Using a combination of classical and operant conditioning, dogs are trained to behave a certain way once they have detected a target odor and are close to the source of that odor. The use of a detection dog and location of one or more of the target odors will typically go as follows: (1) the dog is deployed with a search command. (2) Casting stage: the dog will start sampling the air currents trying to locate an odor plume. As air current carries the target odor, dogs locate the edges of the odor plume and try to find the source. The dog will move its head from side to side trying to locate the edges of the odor plume and the odor source. (3) Localizing stage: once the dog has detected the odor plume, it engages in noticeable changes of behavior. The dog often becomes visibly excited with its body becoming more rigid, changes in rates of breathing, and appear more focused. The dog will close its mouth, very focused sniffing occurs, and sniffing frequency increases. Sometimes the dog will appear to become frantic or engage in a sudden turn of the head. (4) Alert phase: after the dog has successfully located the source of the odor, the dog has been conditioned to perform a specific behavior, often called a trained final response. The most common trained final responses in professional detection work is a sit or freeze and in civilian sport detection is a head turn and look at the handler. The dog is then rewarded with a reinforcer (e.g., food or toy).

Behavioral stages were defined stages that correspond to the search stages explained above: casting, localizing, and alerting. An off-task stage was also added for times where the dog was not engaged in the search task, and any off-task behavior was removed from all analyses. Current literature defines three search phases in dogs (i.e., Thesen et al.^58^: initial, deciding, and tracking phases), where each phase differs from the other based on speed, sniff frequency, and time spent sniffing. The addition and use of distinct casting and localizing stages in this study bridges the gap in terminology from tracking and trailing to the wider olfactory literature^2,11,12,18–22,26,58^. Therefore, the terms used in the current study better classify and describe the search stages dogs iterate through as they navigate the odor plumes they search.

Individual behaviors were defined as distinct behaviors displayed independently of search stage and collected as both the number of incidents and amount of time the behaviors were displayed. The behaviors were selected after observations made during the trials and because they have been linked with psychological and physiological states of being, such as confusion, avoidance, anxiousness, focus, and alertness^58–60^. Twelve individual behaviors were chosen and coded (see SI Table 3 for definitions). All behaviors fell into two major categories of physiological stress: focused/attentive behavior, which can also be classified into the greater category of eustress or distress. In BORIS, search stages were scored as start/stop during the timeline of the search. The individual behaviors were collected as both count data and amount of time the behaviors were displayed. Across the two trial dates, 55 videos were coded twice, once with each ethogram. The video was coded for the entire time the dog was fully visible within the video. There was one trained coder for each ethogram. Once the videos were coded, the two behavior types were paired statistically so that the appropriate individual behaviors fell within the relevant behavioral state according to video timing.

### Statistical analysis

Statistical analyses of search stage and behavior data was performed in R version 4.3.1^56^. Three-dimensional distances of each dog’s nose to the target odor hole were taken from the kinematic data during search and categorized based on the behavior stage on the ethogram. Total times in each behavior state were calculated based on the number of video frames between start and stop codes. Data for comparison were tested for normality with a Shapiro-Wilk test and for significance using Welch’s t-test, Wilcoxon rank-sum test, or signed rank test, noted along with statistical values reported in text. P-values were adjusted for multiple comparisons using a Bonferroni correction. To compare where the dogs concentrated their efforts during search stages, a cluster comparison for two behavior types (casting and localizing) was performed. Clusters were identified with a k-means analysis using the k-means function in base R. K-means clustering is a method that reduces the within cluster variance relative to the clusters’ mean for a user-specified number of groups^61^. To evaluate the accuracy of this clustering scheme for each chemical relative to our expectation we then calculated the gap statistic of each cluster using the function clusGap from cluster package (clusterpkg) was then calculated. The gap statistic summarized the distance of the pooled sum of the squares for a cluster relative to that data under a null distribution^62^. For additional details, see supplementary information.

### Animal use ethics statement

All experimental procedures in this study were approved by the Institutional Animal Care and Use Committee at Florida International University (FIU IACUC protocol #201314). The study observed all federal, state, and local regulations on the use of vertebrate animals in research. All appropriate permissions from institutions and trial locations were obtained to use dogs in research for this study.

### Supplementary Information

Supplementary Information.

## Data Availability

All data and code used to produce results can be found on Github at https://github.com/lindsaywaldrop/odor-plume-search.
