# Supplementary material for: Physical properties of odorants affect behavior of trained detection dogs during close-quarters searches
Source: Sci Rep. 2024 Feb 28;14:4843. doi: 10.1038/s41598-024-55323-y (PMC10902392; doi:10.1038/s41598-024-55323-y)
Supplement: Supplementary file 1 — Supplementary Information. [file 41598_2024_55323_MOESM1_ESM.pdf]

# Supplemental Information to 'Physical properties of odorants affect behavior of trained detection dogs during close-quarters searches'

Daniel Mejia[1], Lydia Burnett[2], Nicholas Hebdon[1], Peter Stevens[3], Alexis Shiber[1], Clay Cranston[1], Lauryn DeGreeff[2], and Lindsay D. Waldrop[1]

2024-02-06

1. Schmid College of Science and Technology, Chapman University
2. Global Forensic and Justice Center and Department of Chemistry and Biochemistry, Florida International University
3. The Scentsable K9, El Cajon, CA

Corresponding author: L.D. Waldrop waldrop@chapman.edu

## Methods

### Materials

Ammonium hydroxide (Sigma-Aldrich) and 2-etyhyl-1-hexanol (Sigma-Aldrich) were selected as the two target odorants of interest due to differences in their physical properties (Table 1).

SI Table 1: Physical properties of the two target odorant chemicals and two distractor chemicals used in the study: molecular weight, diffusion coefficient and vapor pressure.

| Property | 2E1H | $NH_3$ | Bromo-octane | Methyl benzoate |
|---|---|---|---|---|
| Molecular weight (g $mol^{-1}$) | 130.23 | 17.031 | 193.13 | 136.15 |
| Diffusion coefficent ($cm^2$ $s^{-1}$) | 0.0663 | 0.2403 | 0.0591 | 0.0711 |
| Vapor pressure (atm) | 0.000179 | 9.9 | 0.000500 | 0.000447 |

In order to train the two different target odors, handlers were given controlled odor mimic permeation systems (COMPS) as training aids. COMPS are a tool that controls odor availability during training. For this study, each COMPS consists of 20 $\mu$L of analyte pipetted onto a 2 inch by 2 inch piece of cotton-blend gauze pad (Dukal, LLC) which was heat-sealed in a 2 by 3 inch plastic bag. The analyte is released at a rate determined by the thickness and surface area of the bag[1]. In order to equalize the amount of 2E1H and ammonia presented to the canine during training, a 3 MIL low density polyethylene bag (LDPE; Uline) was used 2E1H and a 8 MIL LDPE bag (Uline) was used for ammonia. Non-target or distractor odorants were prepared in a similar way for methyl benzoate (Sigma-Aldrich; 3 MIL bag) and bromooctane (Sigma-Aldrich; 8 MIL bag).

### Participants

Fourteen civilian canines were recruited through the National Association of Canine Scent Work (NACSW)™. NACSW canines train to detect essential oils for competition. Canines recruited from Nosework 3 title or higher. Dogs at this level are trained to detect multiple odor sources, trials with blanks, and in various search situations.

Three small dogs (< 30 lbs) and eleven large dogs ($\geq$ 30 lbs) were included of all different breeds (Table 2). The handlers were not compensated for participation. The study observed all federal, state, and local regulations on the use of vertebrate animals in research (FIU IACUC protocol #201314).

SI Table 2: Dog Participant Information

| Dog number | Breed | Weight (lbs) |
|---|---|---|
| 1 | Laborador retriever | 60 |
| 2 | Shetland Sheepdog | 30 |
| 9 | Belgian tervuren | 51 |
| 11 | Belgian malinois mix | 75 |
| 12 | English shepherd | 65 |
| 14 | Poodle | 38 |
| 15 | Pit bull terrier | 51 |
| 17 | Labrador retriever mix | 70 |
| 19 | Golden retriever | 60 |
| 20 | Boxer | 56 |
| 21 | Bull terrier | 57 |
| 22 | Chihuahua mix | 8 |
| 23 | Dachshund | 8 |
| 24 | Dachshund | 11 |

**Training**

During the training period, handlers logged the time in which they used each COMPS and were instructed to throw away the COMPS and open a new one after it was exposed to the air for a total of six hours. Handler-dog teams were given six weeks to train with the COMPS before participating in the trials. Each handler-dog team trained individually as they would for typical Nosework odor competitions.

**Experimental Setup and Procedure**

The trial was held in Huntington Beach, California in February, 2023 at a dog training facility (The Hounds Grounds). The trial was held during the morning, which was cloudy and rainy (temperature 17°C, 80% relative humidity). All HVAC remained off during the duration of the experiment such that the inside conditions were consistent with the outside conditions in terms of temperature and humidity. The main search area was separated from the front door by a four-foot high wooden wall, which limited much air movement in the main area when the outer doors opened and closed. Temperature, humidity, and wind conditions varied little during the three hours the trials were held.

Two plywood trials walls were set up in the middle of each location (Figure 1). The walls consisted of three four foot by four foot panels of 1/2 inch plywood with two inch by three inch studs that were connected in a C pattern, the center panel of which contained four imprinting holes (3" interior, 4" exterior ABS toilet flange; OATEY) spaced seven inches horizontally apart. One wall was for large dogs where the imprinting holes were placed 16 inches on center from ground; another was for small dogs and had the holes spaced seven inches on center from the ground. Each hole presented a single odorant to the dogs through a small opening on a plastic PVC cap. One COMPS was placed in three of the four holes, containing one of two distracting odorants (either methyl benzoate or bromooctane) or one of the two target odorant (either 2E1H or $NH_3$). A fourth hole had no odor (blank).

COMPS were placed in the imprinting devices five to ten minutes before dogs were allowed to interact with the wall. Dogs were then run consecutively. No special effort was made to reset or control the odor plume from the wall between dogs. After each run with a target odor, distracting-odor COMPS were replaced. The white, PVC cap and ABS imprinting hole were replaced between 2E1H and $NH_3$ to limit cross-contamination between target odors. A 15- to 20-minute period between runs with each target odor was given to allow the previous odor plumes to dissipate.

Dogs were allowed to search a similar imprinting wall with a nose work odor (birch oil) source before and after each experimental run. Each dog was sent to their corresponding wall twice, once where the target odor was $NH_3$ and the other when it was 2E1H. Two observers remained behind the dog and handler dyad while they were sent to the corresponding wall to note observations and to coach the handler, if needed. The handlers were blind to which hole contained the target odor. The handler was instructed to search the wall and indicate if their dog alerted to the location of the odor source. Once the dog had searched the wall and either alerted to the target odor or had worked the wall sufficiently, the dog-handler team was exited the facility. To assess whether the dogs were more successful at locating one odor or another, the successful versus unsuccessful alerts were noted by both observers during each run.

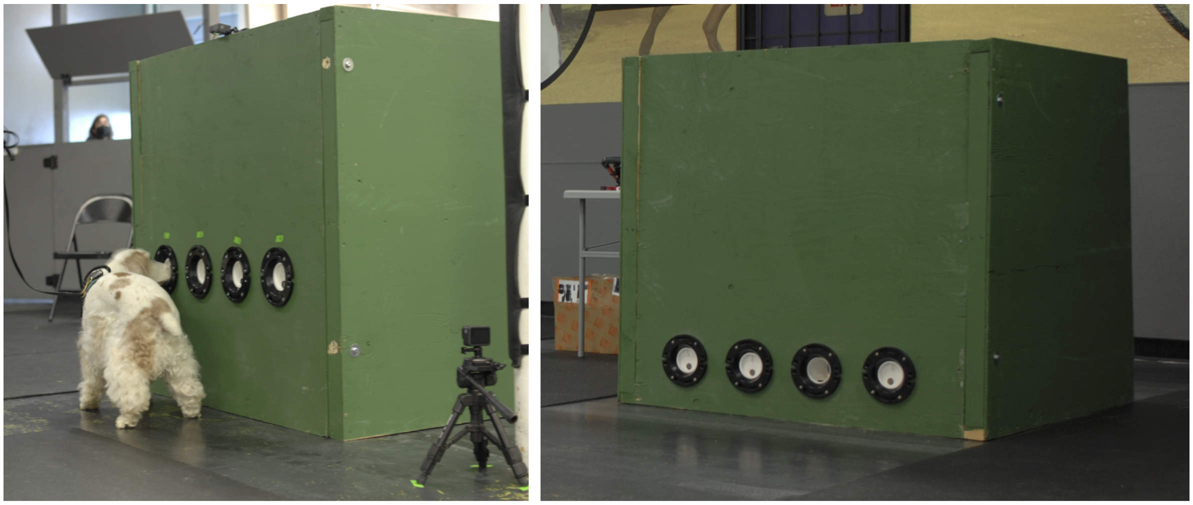

SI Figure 1: Trial walls used for the experiment showing the relative placement of imprinting holes. Left: large wall, right: small wall. Left image shows side and top camera set up for capturing video data. Leashed dogs were allowed to interact with the wall freely. Photos by Monica Moljo.

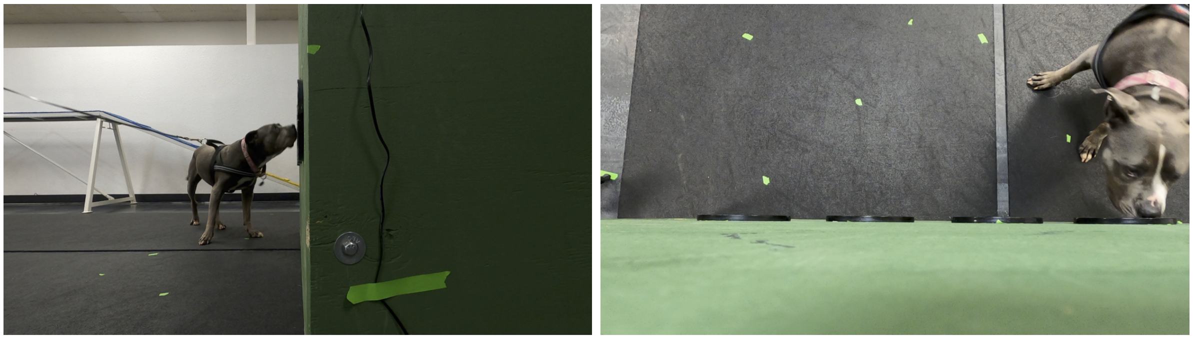

SI Figure 2: Side (left) and top (right) views of the cameras during a search.

Two video cameras (GoPro Hero 8 Black, GoPro, San Mateo, CA) were placed in view of the front of the wall where dogs interacted with odors, one above the holes and one to the right side of the trial wall, resulting in a top view and side view, respectively (Figure 2). Cameras were placed in linear mode using 30 frames per second to record at 4K resolution. Cameras ran continuously for the time that the dogs interacted with the wall area. Videos were calibrated with a three-dimensional calibration object on sight immediately before running dogs in the trial and for each wall (large and small). Cameras were synced using a verbal cue and aligned using iMovie during analysis. Calibration footage was taken each time the cameras were initially set up or moved.

**Behavioral Analysis**

To analyze the behaviors occurring along the search path, two ethograms were created to code behavioral stages related to searching and other, relevant individual behaviors that occurred during the search. Each ethogram was created using the Behavioral Observation Research Interactive Software (BORIS) and coded using the same software[2]. Behavior states and individual behaviors were scored by two trained observers (one for behavior states, one for individual behaviors).

Using a combination of classical and operant conditioning, dogs are trained to behave a certain way once they have detected a target odor and are as close to the source of that odor as they can possibly get. The use of a detection dog and location of one or more of the target odors will typically go as follows:

1. The dog is deployed with a search command.

2. **Casting stage**: the dog will start sampling the air currents trying to locate an odor plume (i.e., "scent cone"). As air current carries the target odor, dogs locate the edges of the odor plume and try to find the source. The dog will move its head from side to side trying to locate the edges of the odor plume and the odor source.
3. **Localizing stage**: once the dog has detected the odor plume, it engages in noticeable changes of behavior. The dog often becomes visibly excited with its bodies becoming more rigid, changes in rates of breathing, and appear more focused. The dog will close its mouth, very focused sniffing occurs, and sniffing frequency increases. Sometimes the dog will appear to become frantic or engage in a sudden turn of the head.
4. **Alert phase**: after the dog has successfully located the source of the odor, the dog has been conditioned to perform a specific behavior, often called a Trained Final Response (TFR). The most common TFR's in professional detection work is a sit or freeze and in civilian sport detection is a head turn and look at the handler. The dog is then rewarded with a reinforcer.

Behavioral stages were defined stages that correspond to the search stages explained above: casting, localizing, and alerting. An off-task stage was also added for times where the dog was not engaged in the search task. The current literature has agreed upon the three defined search phases in dogs defined in Thesen et al.[3] (initial, deciding, and tracking phases), where each phase differs from the other based on speed, sniff frequency, and time spent sniffing. The addition and use of distinct casting and localizing stages in the current study bridges the gap in terminology from tracking and trailing to the wider olfactory literature, as well as remaining consistent with the stages outlined in Thesen et al.[3–12]. Therefore, the terms used in the current study better classify and describe the search stages dogs iterate through as they navigate the odor plumes they search.

Individual behaviors were defined as distinct behaviors displayed independently of search stage and collected as both the number of incidents and amount of time the behaviors were displayed. The behaviors were selected after observations made during the trials and because they have been linked with psychological and physiological states of being, such as confusion, avoidance, anxiousness, focus, and alertness[3,13–17]. Twelve individual behaviors were chosen and coded (see Table 3 for definitions). All behaviors fell into two major categories of physiological stress: focused/attentive behavior, which can also be classified into the greater category of eustress, or distress.

In BORIS, search stages were scored as start/stop during the timeline of the search. The individual behaviors were collected as both count data and amount of time the behaviors were displayed. Across the two trial dates, fifty-five videos were coded twice, once with each ethogram. The video was coded for the entire time the dog was fully visible within the video. There was one trained coder for each ethogram. Once the videos were coded, the two behavior types were paired statistically so that the appropriate individual behaviors fell within the relevant behavioral state according to video timing.

SI Table 3: Table of individual behaviors

| Individual Behaviors | Definitions | States aligned with Behaviors |
|---|---|---|
| Jumping | The dogs jumps, suspending front paws in air or leaning on experimental wall | Distress |
| Look at Handler | Dogs looks at handler | Distress |
| Pawing | Paws any part of the wall or floor | Distress |
| Sniffing | Nose near the wall and clear movement of nose to indicate sniffing | Focused/Attentive |
| Shoulder shrug | Tightening of chest and shoulders so that it appears as if the dog is shrugging | Focused/Attentive |
| Eye Convergence | Both eyes converged on a point on the wall or at the handler, indicating focused and intense staring | Focused/Attentive |
| Facial Tensing | Tensing of the facial muscles | Focused/Attentive |
| Head Turn | Turning the head away from the wall and towards the handler | Distress |
| Lip Lick | Clear tongue protrusion and lip licking | Distress |
| Ear Prick | Muscular modulation of ear to either perked up position or pointed back | Focused/Attentive |
| Head Snap | Clear and quick snap of head back to original head position | Focused/Attentive |
| Tail Wag | Back and forth movement of tail | Focused/Attentive |

### Kinematic Analysis

Movements of dogs during the search were quantified using the tracking software DLTdv8[18]. Two points on the dogs' heads (the middle of the tip of the nose, in between the dogs' eyes) were digitized by hand in each frame of the two synced video views in DLTdv8. The two dimensional points from each video view were then used with easyWand calibration to calculate the three-dimensional position of each digitized point[19]. Three-dimensional points were then exported for further analysis using custom software in R version 4.3.1[20] (See data availability for location of code used for analyses). The same two coders for the behavioral analysis completed the kinematic analysis, with one coder acting as a confirmatory judge of the first coder's analysis.

### Statistical analysis

Statistical analyses of search stage and behavior data was performed in R version 4.3.1[20]. Data for comparison were tested for normality with a Shapiro-Wilk test and for significance using Welch's t-test, Wilcoxon rank-sum test, or signed rank test, noted along with statistical values reported below. P-values were adjusted for multiple comparisons using a Bonferroni correction.

To compare where the dogs concentrated their efforts during different types of behavior, a cluster comparison for two behavior types (casting and localizing) was performed. Clusters were identified with a k-means analysis using the k-means function in base R. K-means clustering is a method that reduces the within cluster variance relative to the clusters' mean for a specified number of groups[21]. K-means quantification requires the user to specify the number of clusters to be found. In this analysis a cluster would equate to a high density zone of a given behavior. For our experimental configuration this was set to five, one per odor hole and one additional for background/off-wall behavior.

To evaluate the accuracy of this clustering scheme for each chemical relative to our expectation we then calculated the gap statistic of each cluster using the function `clusGap` from cluster package was then calculated[22]. The gap statistic summarized the distance of the pooled sum of the squares for a cluster relative to that data under a null distribution[23]. The higher the gap statistic, the more defined a cluster is. Gap statistics are often used to search for the optimal number of clusters in the system but here were used to

compare the performance of a single clustering configuration for each target chemical to assess how centralized the behaviors of the dogs are in their search.

### Full reproduction of analysis included in the main manuscript and supplemental infomration documents

The analysis for this study was completed in RStudio version 2023.06.0+421 (2023.06.0+421) using the R Markdown and R Bookdown packages in fully reproducible code notebooks (RMD files). All figures and analyses are available in the Github repository: https://github.com/lindsaywaldrop/odor-plume-search. Detailed instructions can be found in the README file.

In order to reproduce these documents, use RStudio to open a new project based on version control and clone the project using the repository's web URL. Alternatively, download the code release indicated on the Github repository's site, expand the compressed file, and open the included .Rproj file in RStudio. There are a number of packages to install, which can be installed using the install-required-pkgs.R script in the src/ folder. Then, open the main-manuscript.Rmd or supplementary-info.Rmd files in the doc/ folder and knit.

## Results

### Additional Analysis: Before and after training on target odors

Dogs in the study were previously trained on NACSW nose work odors which are three essential oils (birch, anise, clove) and were well versed in searching for these target odors before the study. However, no dogs except one had previous training with the two target odors in the study (2E1H and $NH_3$). During the study, dogs were tested on the wall before training for the study's target odors ("untrained") and then after six weeks of training at home ("trained").

The following figures contrast the results of kinematic and behavior analyses between untrained and trained dogs. Note that the sample size for each varies because two dogs in the initial trial were unable to participate in the final trial. Additionally, not all dogs engaged in every phase of search behavior during their runs, leading to different sample sizes between bars.

Cluster plots of untrained and trained dogs in casting shows that patterns of movement are similar between 2E1H and $NH_3$ before training (Fig. 3a, b) and show much more spread movements after training (Fig. 3c, d). k-mean gap scores are low, (range: 0.6, 0.83) with the highest scores for 2E1H untrained tracks.

For localizing, there are large and obvious differences between untrained and trained trial runs (SI Fig. 4). Many of the dogs before training did not engaging in localizing behaviors at all, with only two and three dogs engaging in search behavior in the kinematic search area. There are no significant differences in distance from the target-odor source before training (SI Fig. 5a) but significant differences after training (SI Fig. 5c).

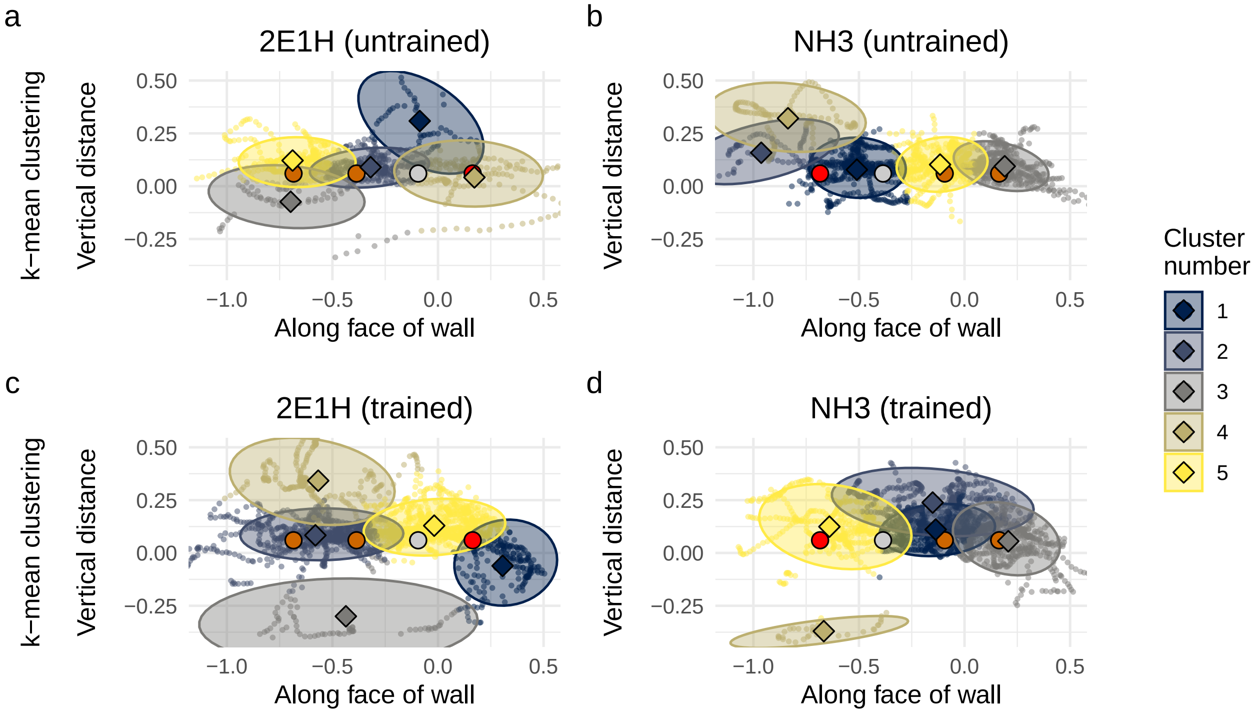


SI Figure 3: Point-density plots (a and b) and k-means clustering plots (c and d) of the front view of trial wall during casting stage for 2E1H (a, c) and $NH_3$ (b, d) for only successful searches. Location of imprinting source holes marked with colored circles (red indicates location of target odorant, blue are distractors, gray is blank).

### Additional Analysis: Removing unsuccessful searches from density and k-mean plots

It is possible that the searches of successful dogs were significantly different than unsuccessful searches, or that these patterns may reveal some important insight to the problem of how searching with success may affect the kinematic analysis from the main manuscript. To investigate these, we removed unsuccessful searches from the k-means analysis and the following visualizations.

SI Fig. 6 show the density maps of casting across all runs for both target odors for successful searches only. Similar to the main manuscript's analysis, casting occurred in a similar way, although successful searches for 2E1H tended to be vertically lower than the density of points from all searches (Fig. 6), although dogs were found to sniff below the holes more for $NH_3$ than 2E1H. K-mean gap scores for clusters were overall lower for both 2E1H (0.21 to 0.55) and $NH_3$ (0.69 to 0.73) than the k-mean gap scores run with all searches. Since there were only two successful searches for 2E1H, this limits the reliability of the k-mean gap scores.

Like the full data set, SI Fig. 7 reflects the same smaller search area in the localizing point density of 2E1H, with the searching highly localized to the holes of the wall and little deviation from the wall's face. Clustering scores correspond to the four source holes of the wall (Fig. 7C) with gap scores of 0.42, 0.66, 0.78, 0.65, 0.76. In contrast, $NH_3$ localizing, while close to the surface of the wall, was spread both above and below the holes, showing clusters that were less distinct and not associated with individual holes (gap scores: 0.78 to 0.77).

Although there are some superficial differences, removing the successful searches point to the same patterns of movement of dogs engaged in the search.

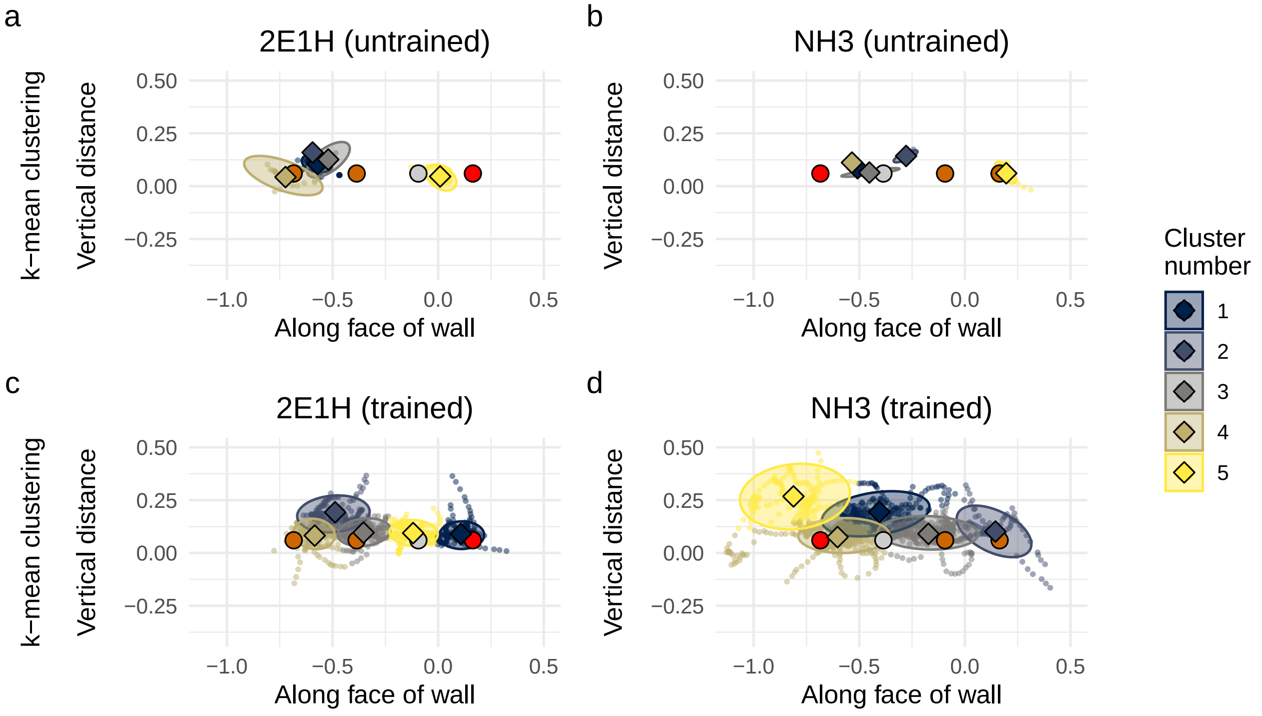


SI Figure 4: Point-density plots (a and b) and k-means clustering plots (c and d) of the front view of trial wall during localizing stage for 2E1H (a, c) and $NH_3$ (b, d) for only successful searches. Location of imprinting source holes marked with colored circles (red indicates location of target odorant, blue are distractors, gray is blank).

### Additional Analysis: Removing unsuccessful dogs (12, 14, and 24) from statistical analysis

Since some dogs were unsuccesssful during searches for both target chemicals, there is a possibility that they behaved differently than dogs that were successful during searches. In order to investigate this question, we re-ran all statistical analyses without these three dogs. These updated statistical analyses do not change the significance of any comparison of the main manuscript. SI Fig. 8 is the main manuscript's Fig. 3 and SI Fig. 9 corresponds to the main manuscript's Fig. 4 with the three unsuccessful dogs removed. For brevity, we will summarize the changes in comparisons we found significant in the full data set:

- The difference in the distance of the nose to the target odor source was significant during casting. Welch Two Sample t-test for the full-set means: 0.619 m and 0.461 m for 2E1H and $NH_3$, respectively; $t = -3.41$; $p = 0.0096$). For the set without 12, 14, and 24: 0.62 m and 0.458 m for 2E1H and $NH_3$, respectively; $t = -2.807$; $p = 0.048$).
- The difference in the distance of the nose to the target odor source was also significant between casting and localizing for $NH_3$. Welch Two Sample t-test for the full-set means: 0.62 m and 0.27 m, for casting and localizing, respectively; $t = 6.77$, $p = 2.1 \times 10^{-6}$. For the set without 12, 14, and 24 means: 0.62 m and 0.27 m, for casting and localizing, respectively; $t = 5.513$, $p = 1.2 \times 10^{-4}$.
- Dogs also spent a longer time engaged in localizing for $NH_3$ than 2E1H. Welch Two Sample t-test for the full-set means: 7.09 s, 3.72 s, $p = 0.048$). For the set without 12, 14, and 24 means: 7.32 s, 3.64 s, $p = 0.042$).
- Dogs spent significantly less time localizing 2E1H than casting for it. Welch Two Sample t-test for the full-set means: 3.72 s, 12.16 s, $p = 0.007$). For the set without 12, 14, and 24: 3.64 s, 11.27 s, $p = 0.007$).

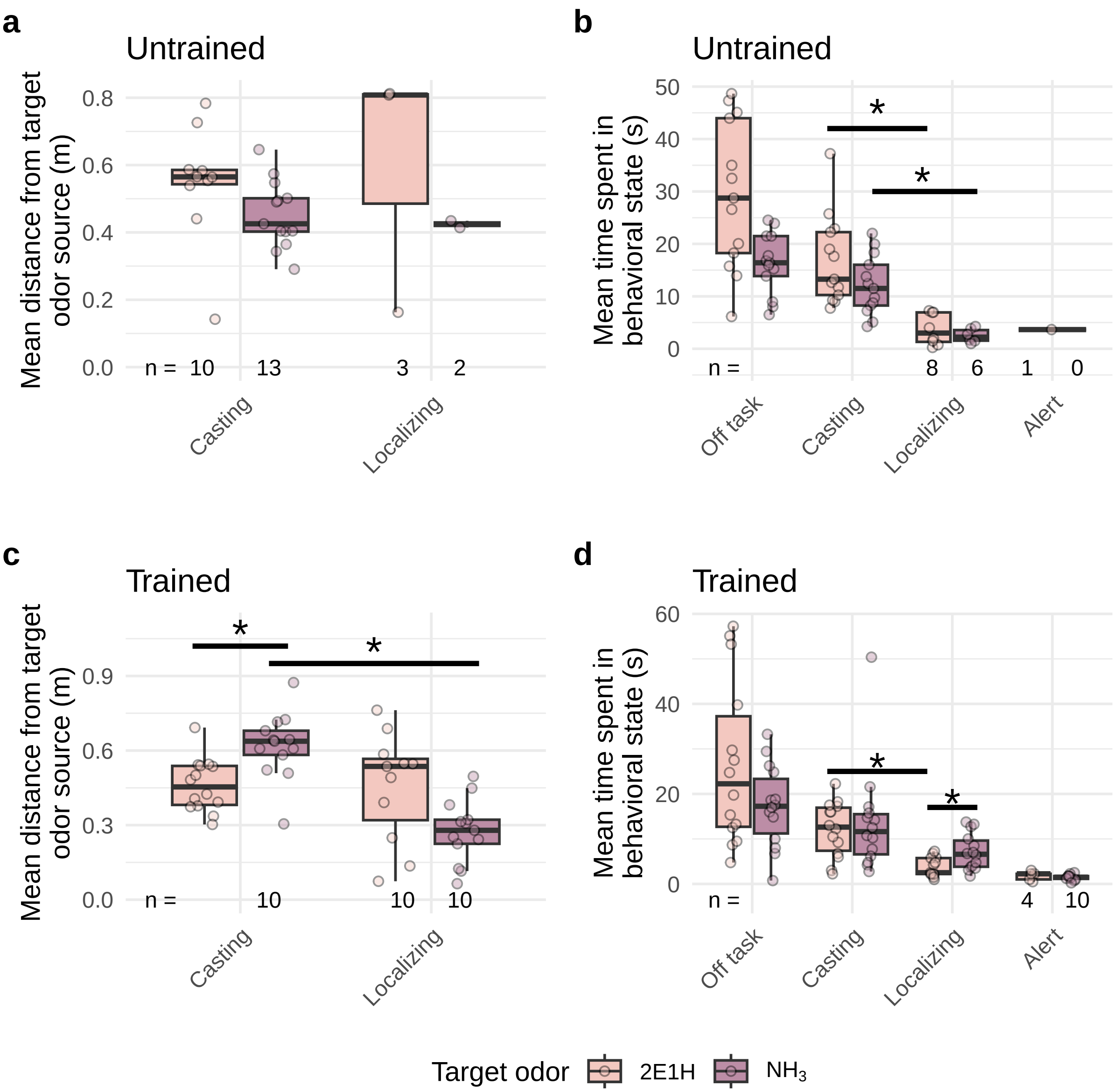


SI Figure 5: Mean distance the dogs occupied in the casting and localizing stages of search (a,c) as well as the mean time they spent within 10 cm of the source of each chemical (b,d) for dogs before training (a, b) and after six weeks of training (c, d). Single black filled circles represent outliers to the boxplot. Sample size is recorded if other than n = 11. * represents a comparison where the $p < 0.05$.

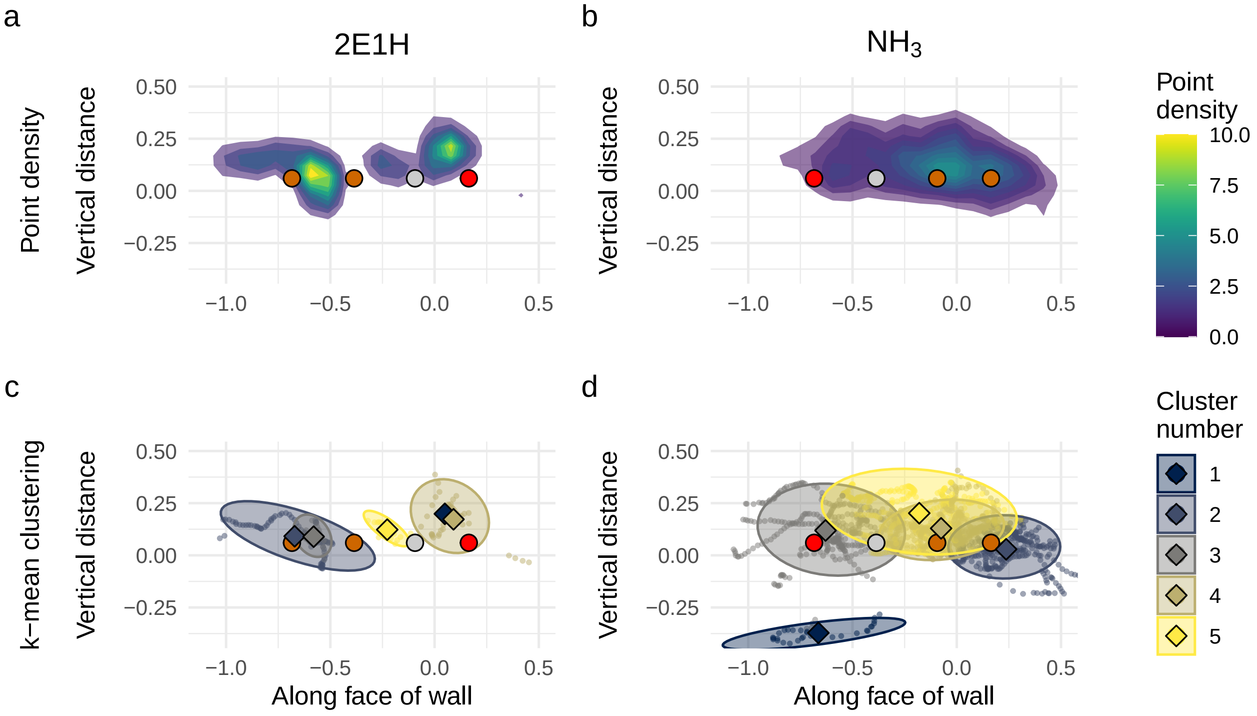


SI Figure 6: Point-density plots (a and b) and k-means clustering plots (c and d) of the front view of trial wall during casting stage for 2E1H (a, c) and $NH_3$ (b, d) for only successful searches. Location of imprinting source holes marked with colored circles (red indicates location of target odorant, blue are distractors, gray is blank).

### Additional Analysis: Comparing tracks for dually successful dogs (11 and 23)

Two dogs (dogs 11 and 23) succeeded in alerting to both target odors. Including only two tracks for each target chemical leads to large variability in k-mean gap scores that are not reflective of a larger sample size of track points since each individual track become clearly visible. This limits the reliability of the gap scores and statistical analysis.

Qualitatively, the larger pattern of movement indicates that both casting and localizing occurred below the line of imprinting holes for both dogs. SI Figs. 10 shows casting and 11 shows localizing for dogs 11 and 23.

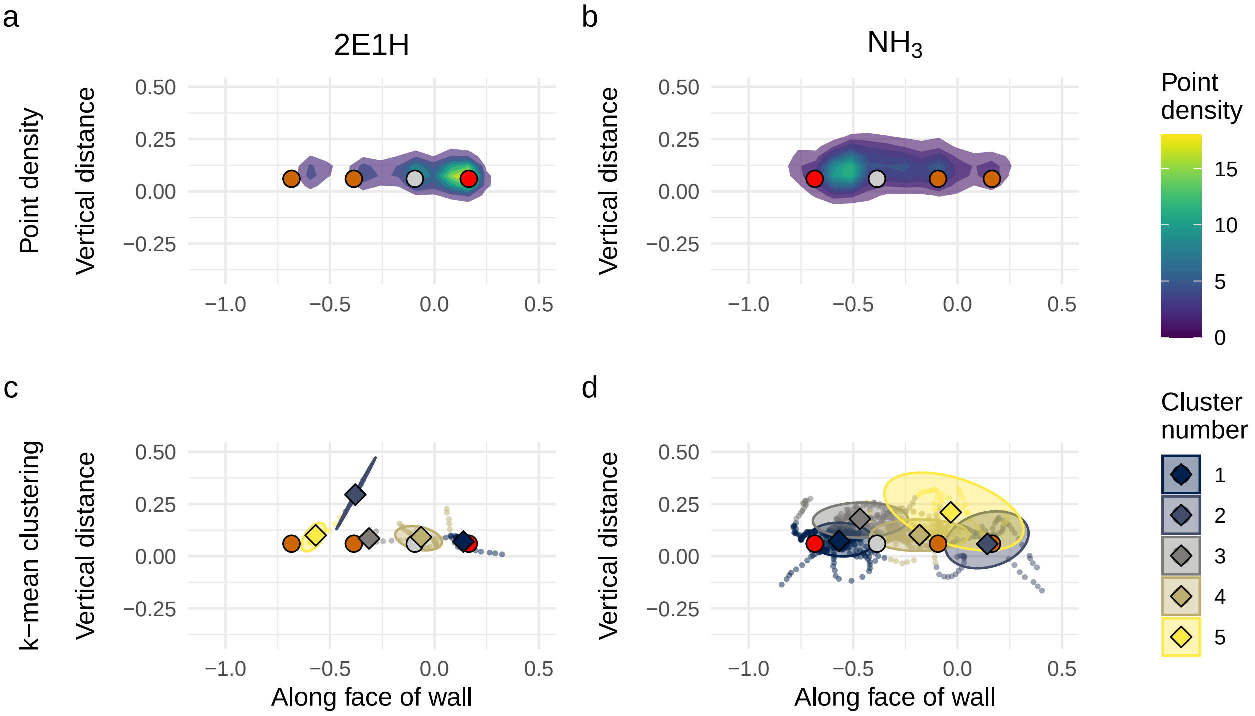


SI Figure 7: Point-density plots (a and b) and k-means clustering plots (c and d) of the front view of trial wall during localizing stage for 2E1H (a, c) and $NH_3$ (b, d) for only successful searches. Location of imprinting source holes marked with colored circles (red indicates location of target odorant, blue are distractors, gray is blank).

### Additional Analysis: Visualizing tracks of individual searches

SI Figs. 12 through 25 present the individual kinematic tracks of the nose for each chemical. The shape of points indicate the task that the dog was engaged in at the time. The color of the path and points progresses from dark blue (at the start) to light blue (at the end) of the track in time. All points within the run are connected, leading to some discontinuity with large lines between sections of the wall. In this case, the dogs often leave the frame of the video and then return at a different position.

Each figure includes the dog's number, runs for each target chemical, and a note on if the search was successful (dog displayed final alert behavior on the correct source hole) or unsuccessful.

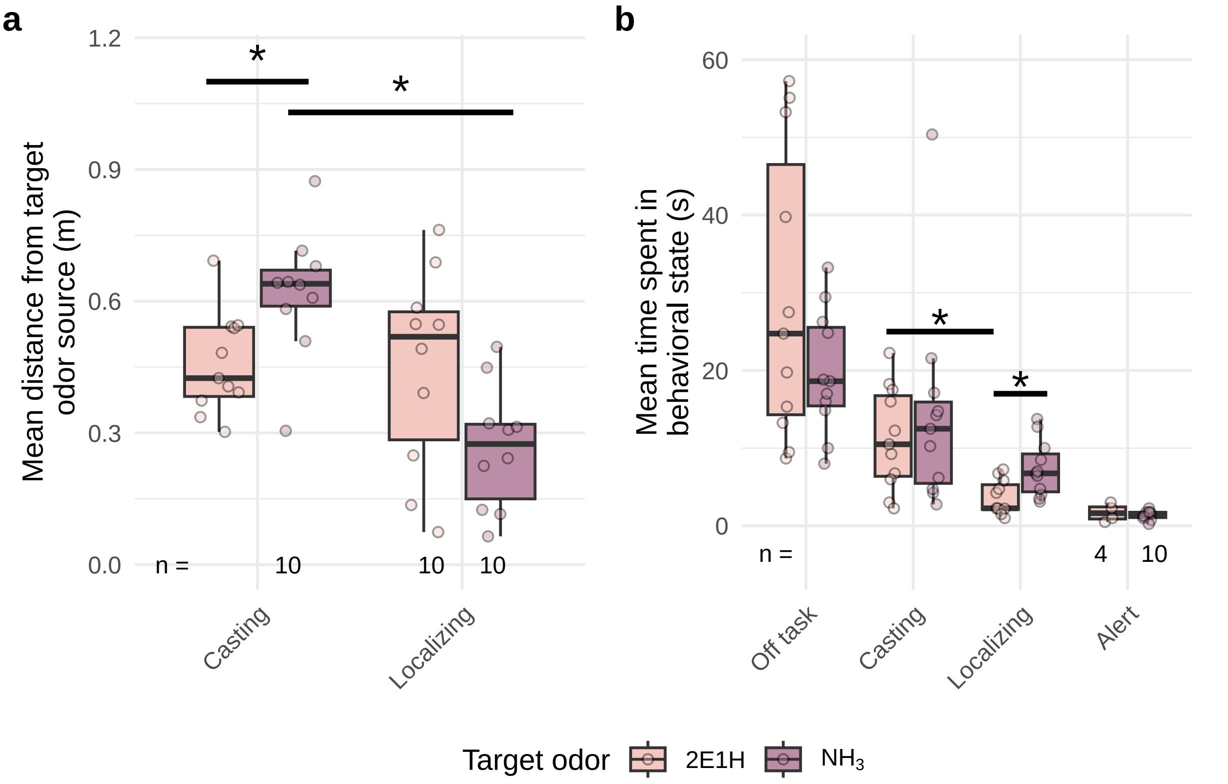


SI Figure 8: Mean distance the dogs occupied in the casting and localizing stages of search (a) as well as the mean time they spent within 10 cm of the source of each chemical (b) excluding dogs 12, 14, and 24. Single black filled circles represent outliers to the boxplot. Sample size is recorded if other than n = 11. * represents a comparison where the $p < 0.05$.

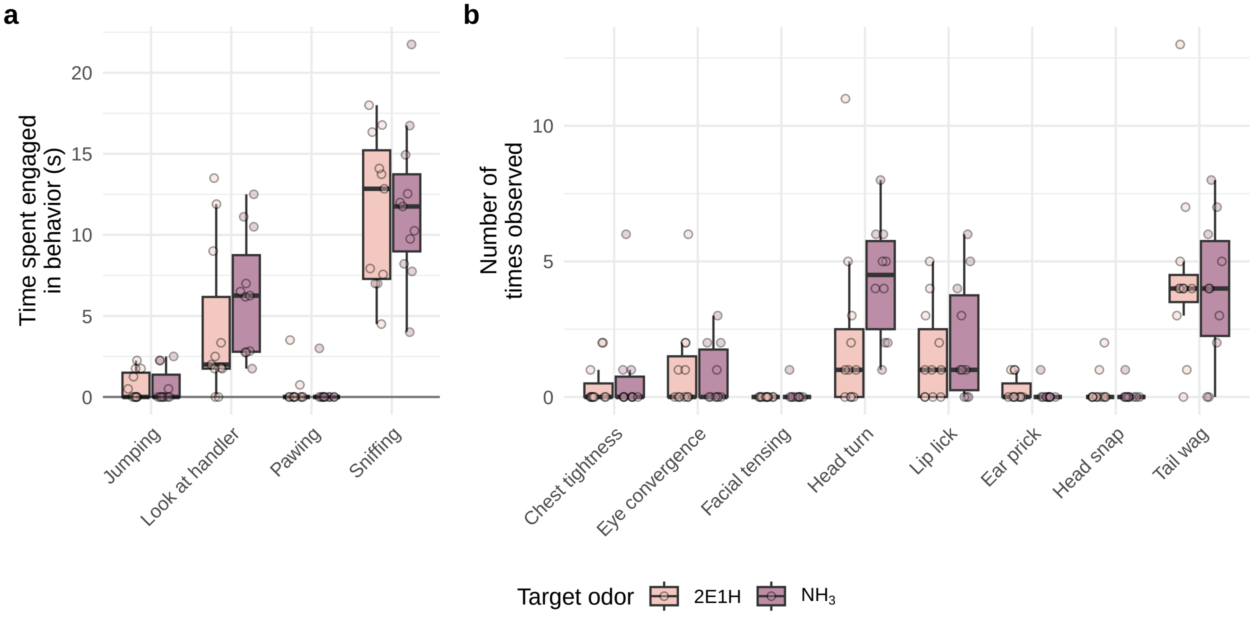


SI Figure 9: Mean time spent showing each of four behaviors (a) and the number of times the dogs displayed each of eight other behaviors (b) for all dogs except 12, 14, and 24.

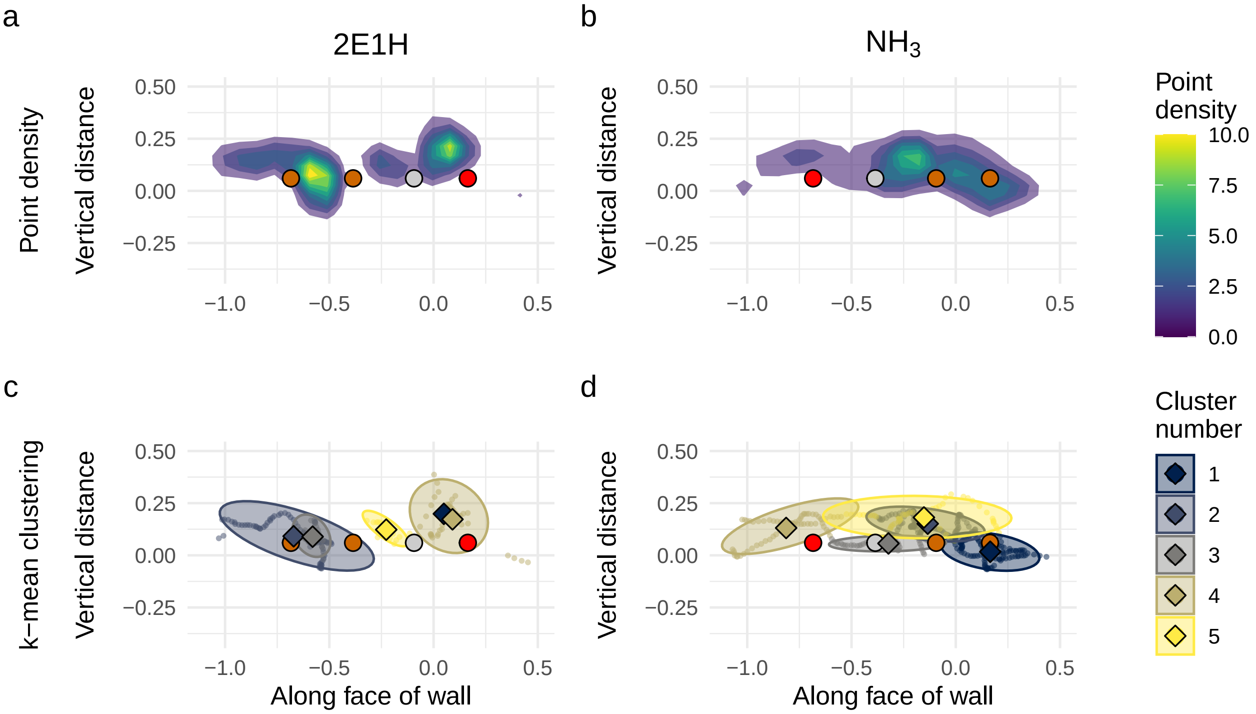


SI Figure 10: Point-density plots (a and b) and k-means clustering plots (c and d) of the front view of trial wall during casting stage for 2E1H (a, c) and $NH_3$ (b, d) for only dogs 11 and 23. Location of imprinting source holes marked with colored circles (red indicates location of target odorant, blue are distractors, gray is blank).

## References


1. Simon, A. G. *et al.* A method for controlled odor delivery in olfactory field-testing. *Chemical Senses* **44**, 399–408 (2019).
2. Friard, O. & Gamba, M. BORIS: A free, versatile open-source event-logging software for video/audio coding and live observations. *Methods in Ecology and Evolution* **7**, 1325–1330 (2016).
3. Thesen, A., Steen, J. B. & Dóving, K. B. Behaviour of dogs during olfactory tracking. *Journal of Experimental Biology* **180**, 247–251 (1993).
4. Weissburg, M. J. & Zimmer-Faust, R. K. Odor plumes and how blue crabs use them in finding prey. *Journal of Experimental Biology* **197**, 349–375 (1994).
5. Settles, G. S. Sniffers: Fluid-dynamic sampling for olfactory trace detection in nature and homeland security – the 2004 freeman scholar lecture. *Journal of Fluids Engineering* **127**, 189–218 (2005).
6. Belanger, J. H. & Willis, M. A. Adaptive control of odor-guided locomotion: Behavioral flexibility as an antidote to environmental Unpredictability1. *Adaptive Behavior* **4**, 217–253 (1996).
7. Hepper, P. G. & Wells, D. L. How many footsteps do dogs need to determine the direction of an odour trail? *Chemical Senses* **30**, 291–298 (2005).
8. Farkas, S. & Shorey, H. Chemical trail-following by flying insects: A mechanism for orientation to a distant odor source. *Science* **178**, 67–68 (1972).
9. Prada, P. A. & Furton, K. G. Birds and dogs: Toward a comparative perspective on odor use and detection. *Frontiers in Veterinary Science* **5**, 188 (2018).
10. Murlis, J., Elkinton, J. S. & Carde, R. T. Odor plumes and how insects use them. *Annual review of entomology* **37**, 505–532 (1992).

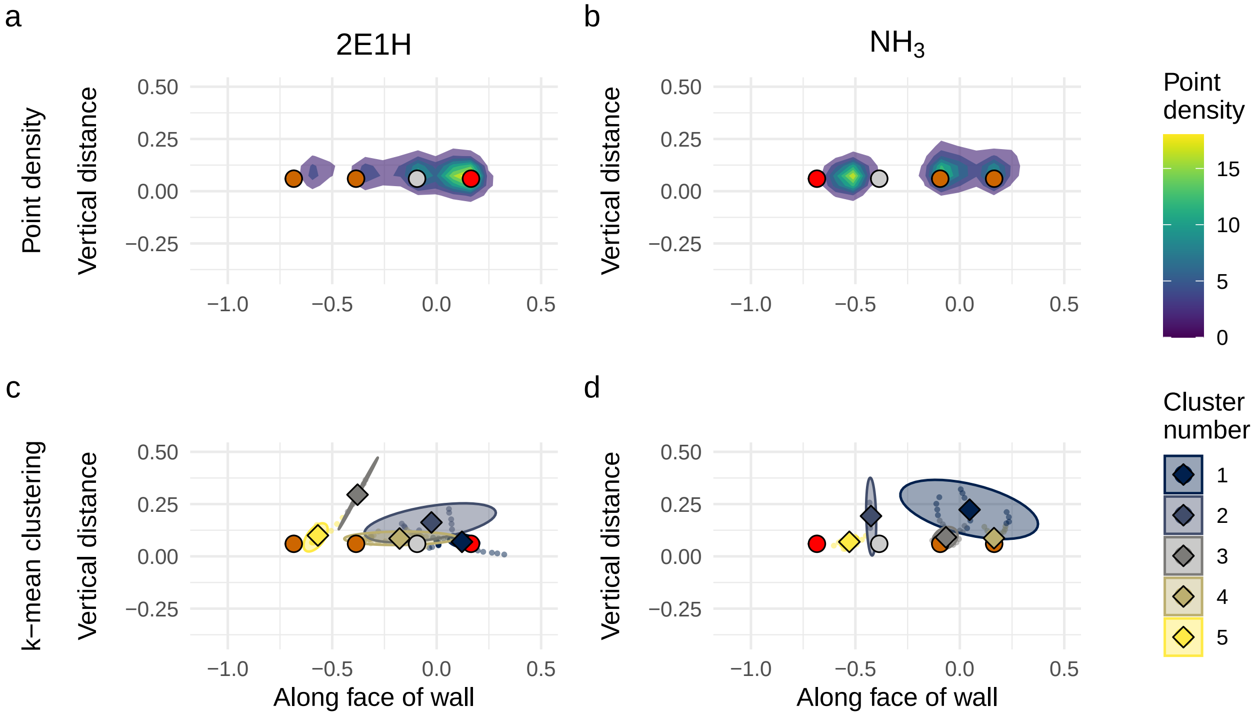


SI Figure 11: Point-density plots (a and b) and k-means clustering plots (c and d) of the front view of trial wall during localizing stage for 2E1H (a, c) and $NH_3$ (b, d) for only dogs 11 and 23. Location of imprinting source holes marked with colored circles (red indicates location of target odorant, blue are distractors, gray is blank).

11. Gire, D. H., Kapoor, V., Arrighi-Allisan, A., Seminara, A. & Murthy, V. N. Mice develop efficient strategies for foraging and navigation using complex natural stimuli. *Current Biology* **26**, 1261–1273 (2016).

12. Reddy, G., Murthy, V. N. & Vergassola, M. Olfactory sensing and navigation in turbulent environments. *Annual Review of Condensed Matter Physics* **13**, 191–213 (2022).

13. Bodnariu, A. *et al.* Indicators of stress and stress assessment in dogs. *Lucr Stiint Med Vet* **41**, 20–26 (2008).

14. Melco, A. L., Goldman, L., Fine, A. H. & Peralta, J. M. Investigation of physiological and behavioral responses in dogs participating in animal-assisted therapy with children diagnosed with attention-deficit hyperactivity disorder. *Journal of Applied Animal Welfare Science* **23**, 10–28 (2020).

15. Beerda, B., Schilder, M. B., Van Hooff, J., Vries, H. W. de & Mol, J. Behavioural and hormonal indicators of enduring environmental stress in dogs. *Animal welfare* **9**, 49–62 (2000).

16. Sinn, D. L., Gosling, S. D. & Hilliard, S. Personality and performance in military working dogs: Reliability and predictive validity of behavioral tests. *Applied Animal Behaviour Science* **127**, 51–65 (2010).

17. Hasegawa, M., Ohtani, N. & Ohta, M. Dogs' body language relevant to learning achievement. *Animals* **4**, 45–58 (2014).

18. Hedrick, T. L. Software techniques for two-and three-dimensional kinematic measurements of biological and biomimetic systems. *Bioinspiration & biomimetics* **3**, 034001 (2008).

19. Theriault, D. H. *et al.* A protocol and calibration method for accurate multi-camera field videography. *Journal of Experimental Biology* **217**, 1843–1848 (2014).

20. R Core Team. *R: A Language and Environment for Statistical Computing.* (R Foundation for Statistical Computing, Vienna, Austria, 2022).

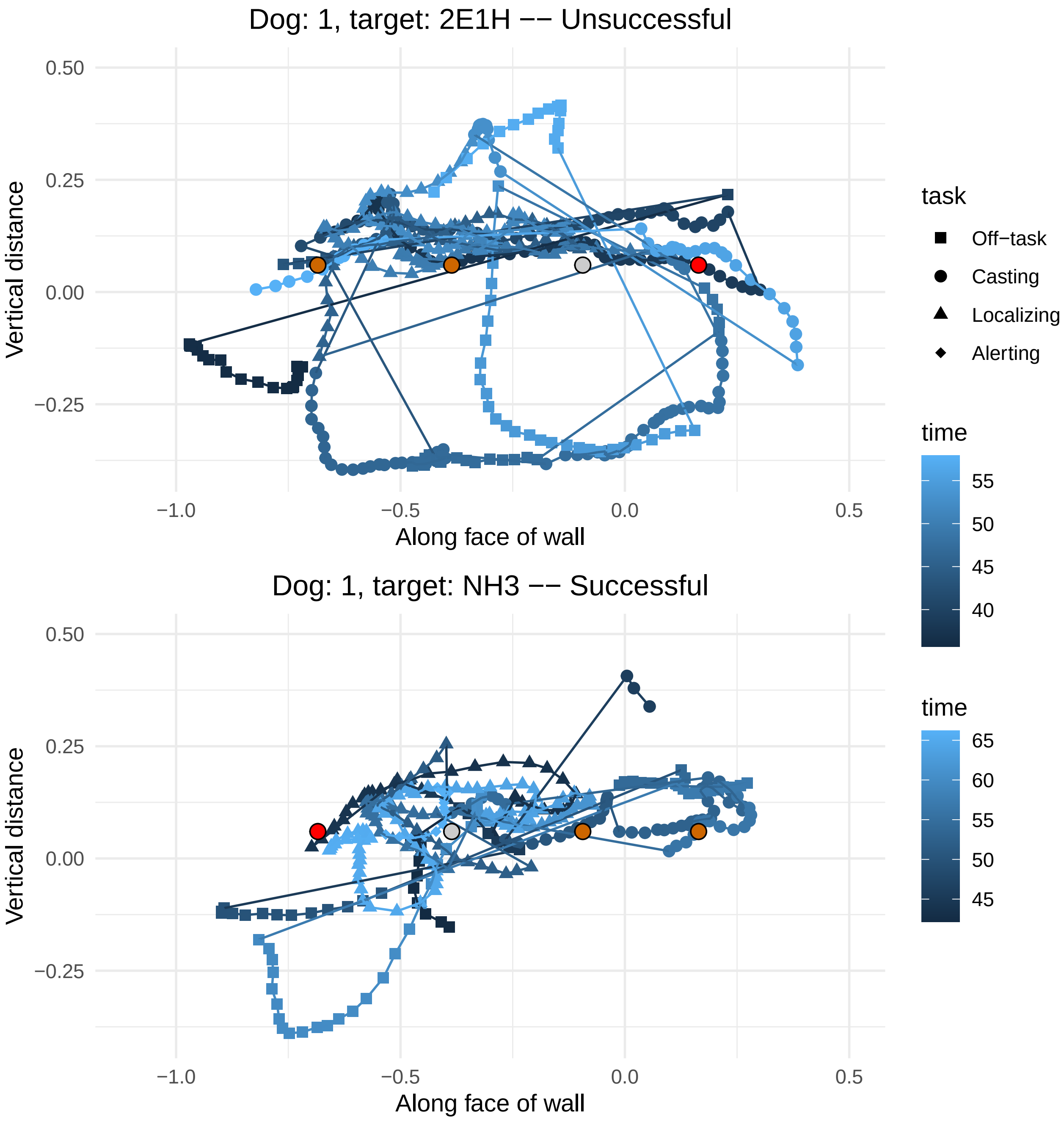


SI Figure 12: Kinematic tracks of dog 1 for both target chemicals.

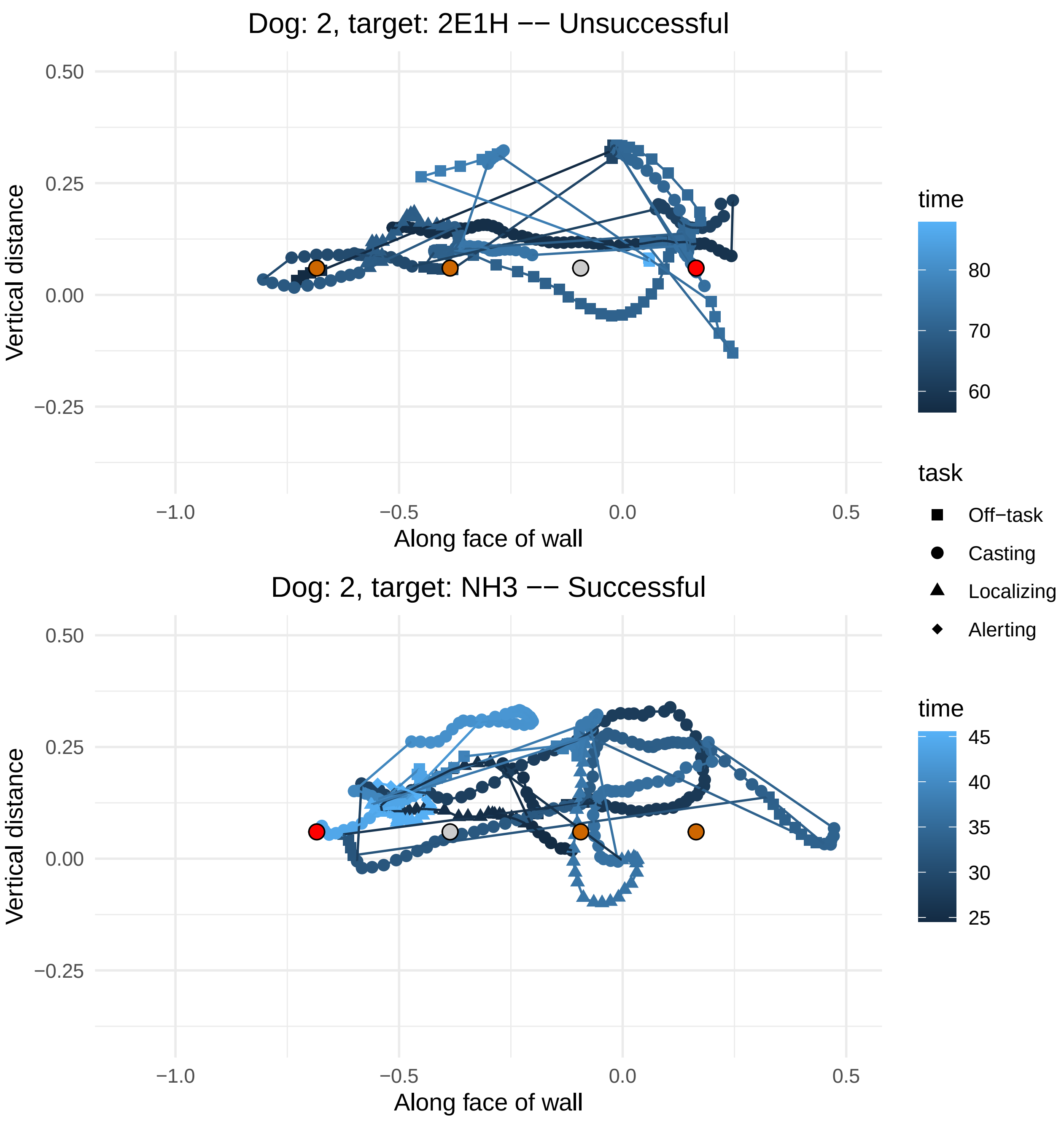


SI Figure 13: Kinematic tracks of dog 2 for both target chemicals.

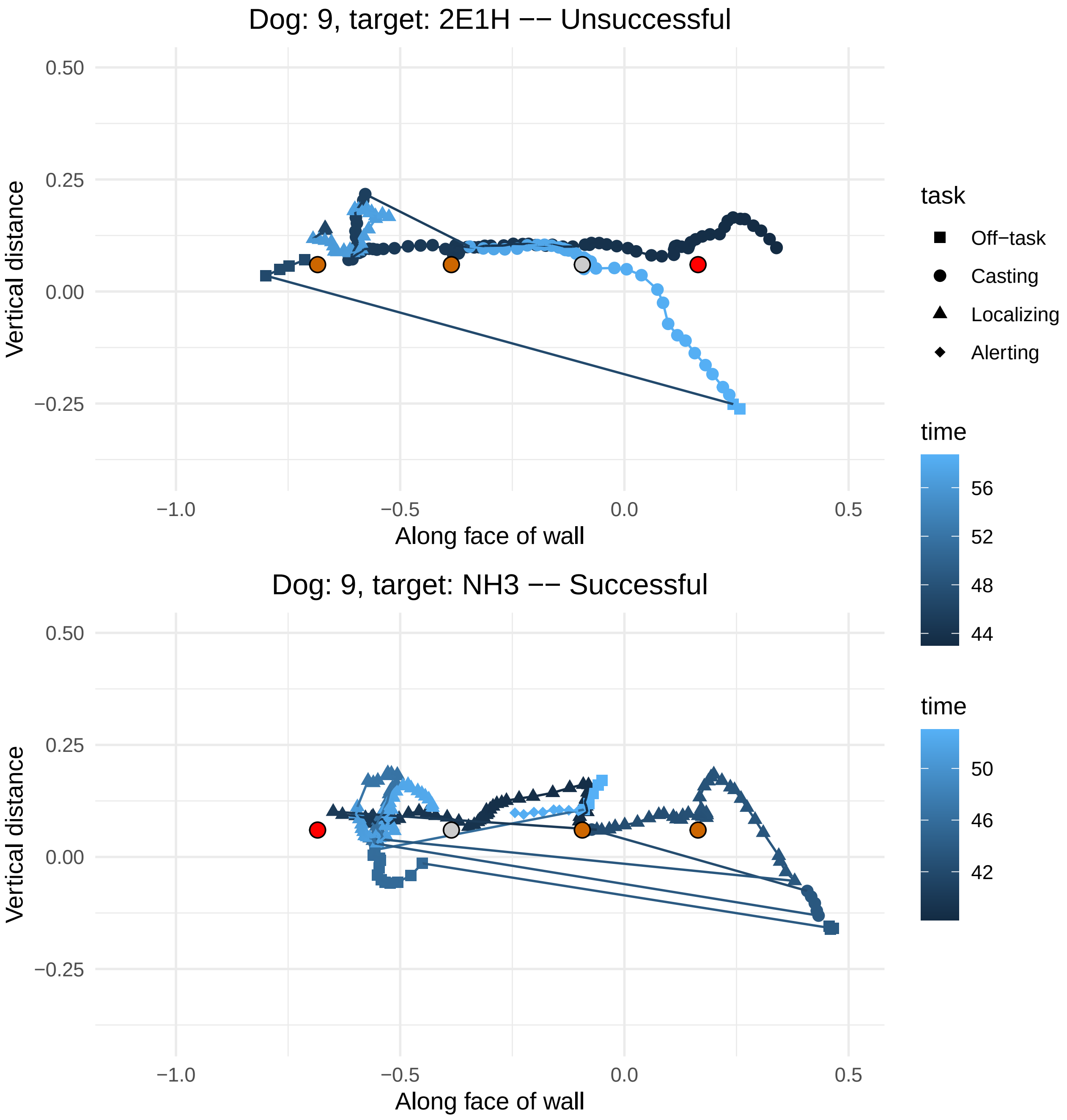


SI Figure 14: Kinematic tracks of dog 9 for both target chemicals.

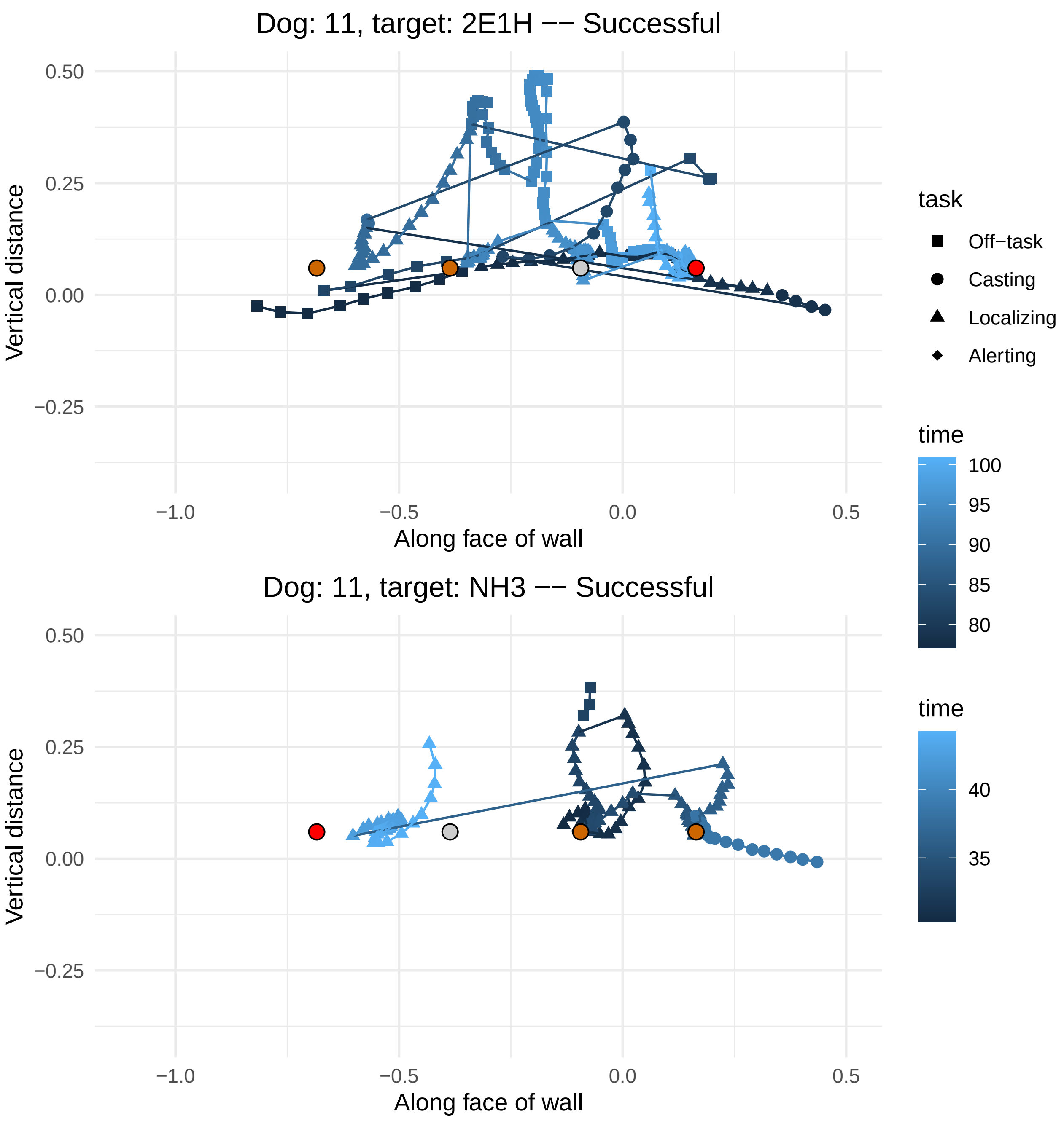


SI Figure 15: Kinematic tracks of dog 11 for both target chemicals.

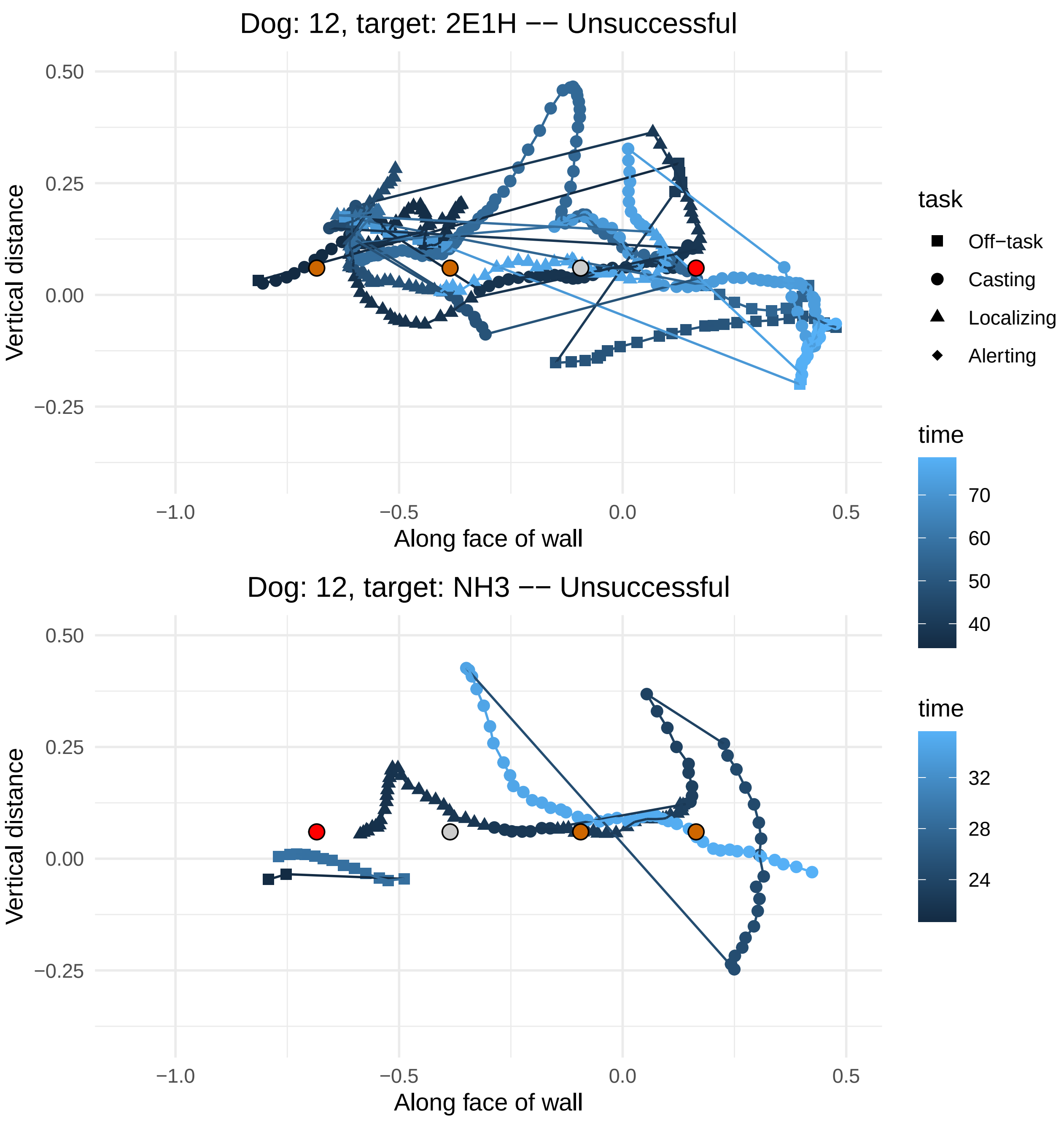


SI Figure 16: Kinematic tracks of dog 12 for both target chemicals.

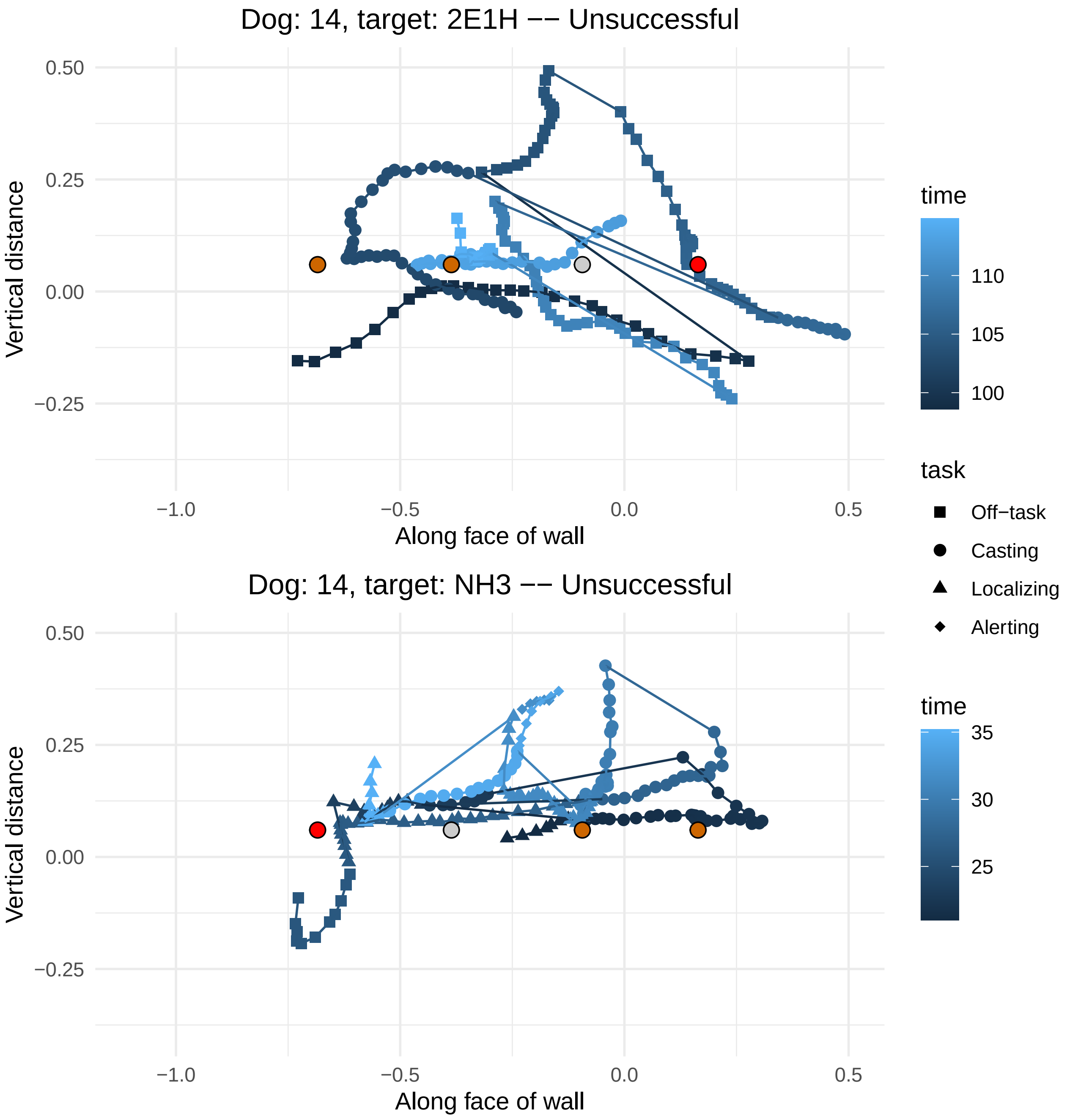


SI Figure 17: Kinematic tracks of dog 14 for both target chemicals.

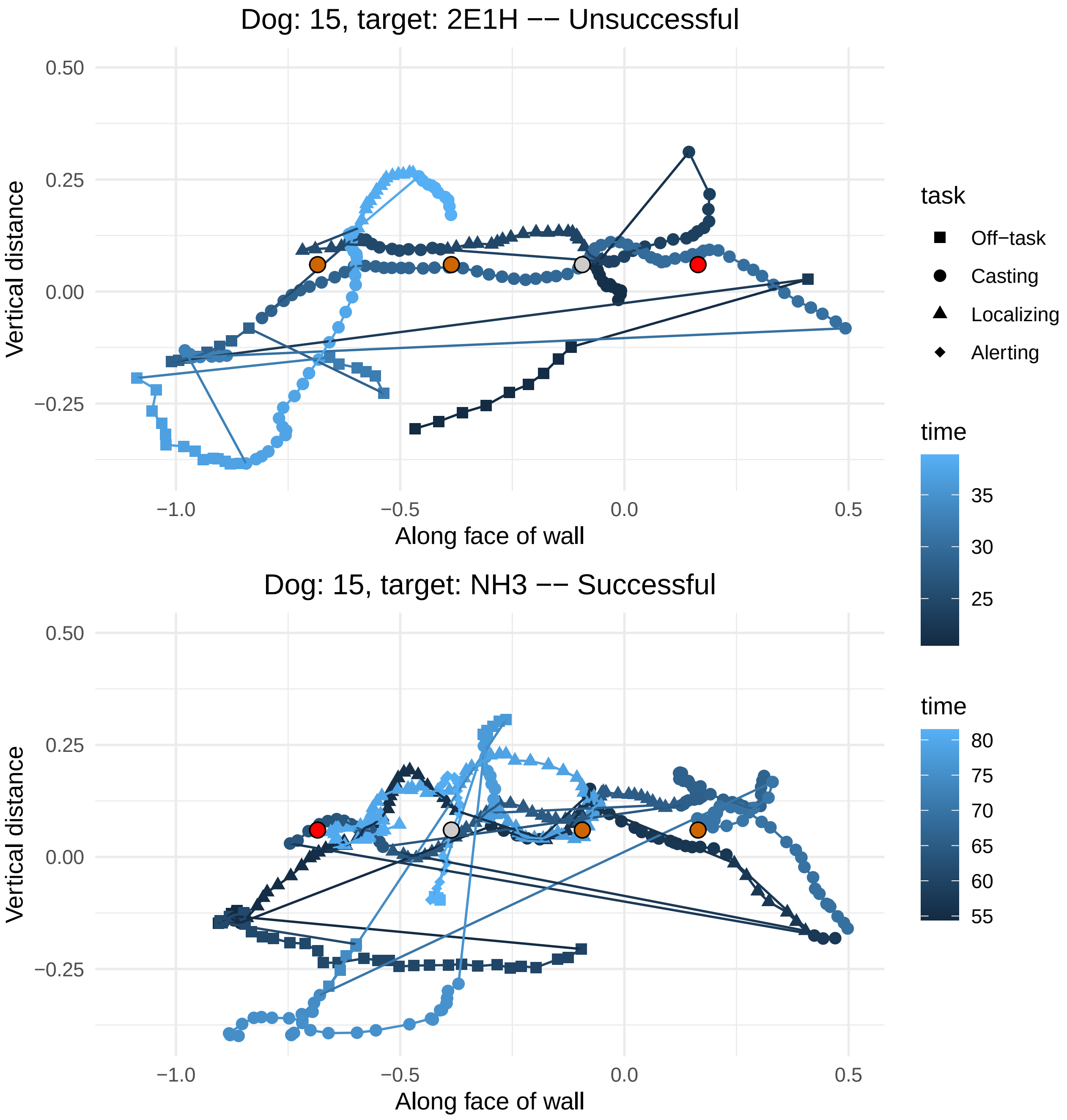


SI Figure 18: Kinematic tracks of dog 15 for both target chemicals.

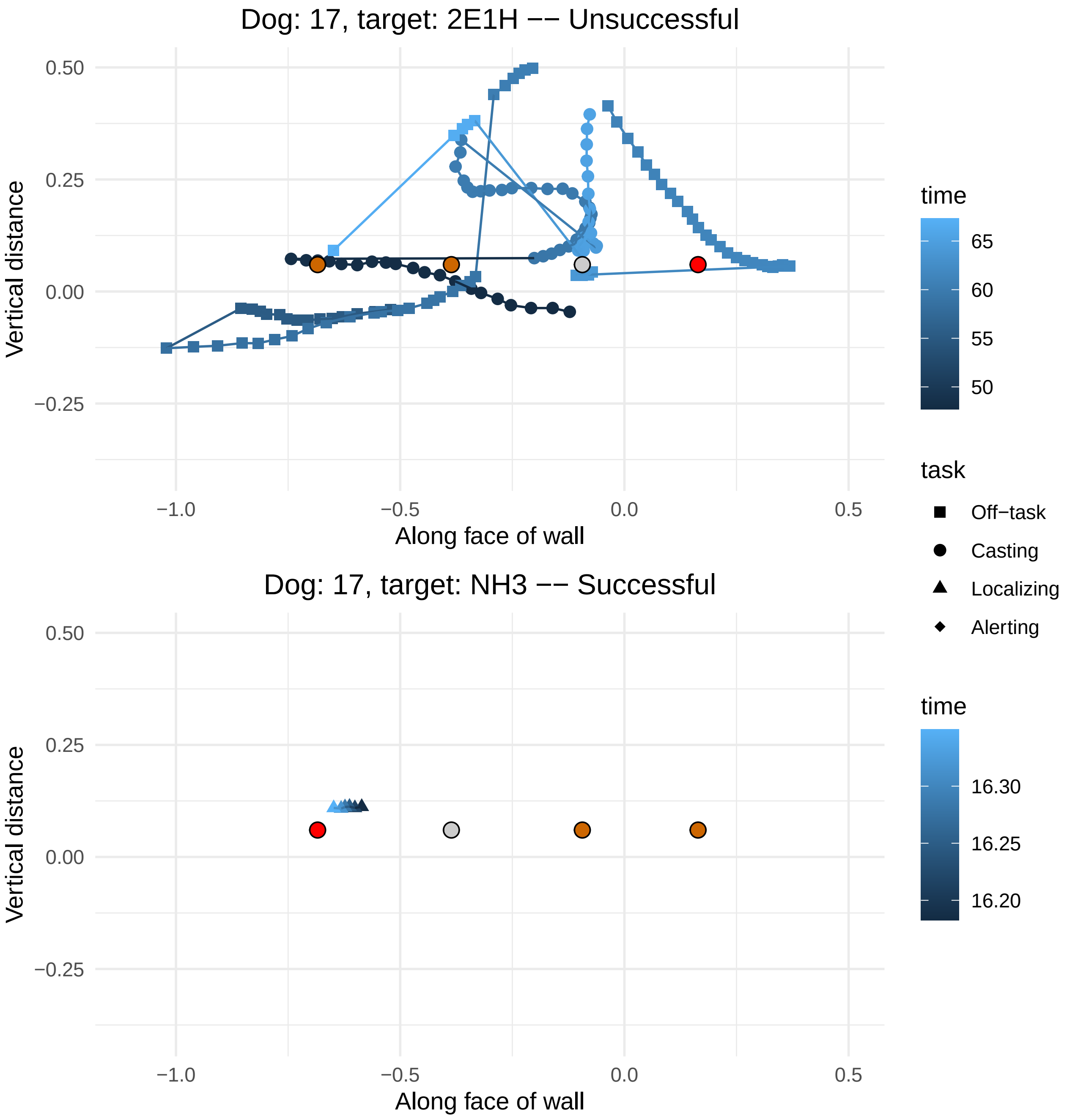


SI Figure 19: Kinematic tracks of dog 17 for both target chemicals.

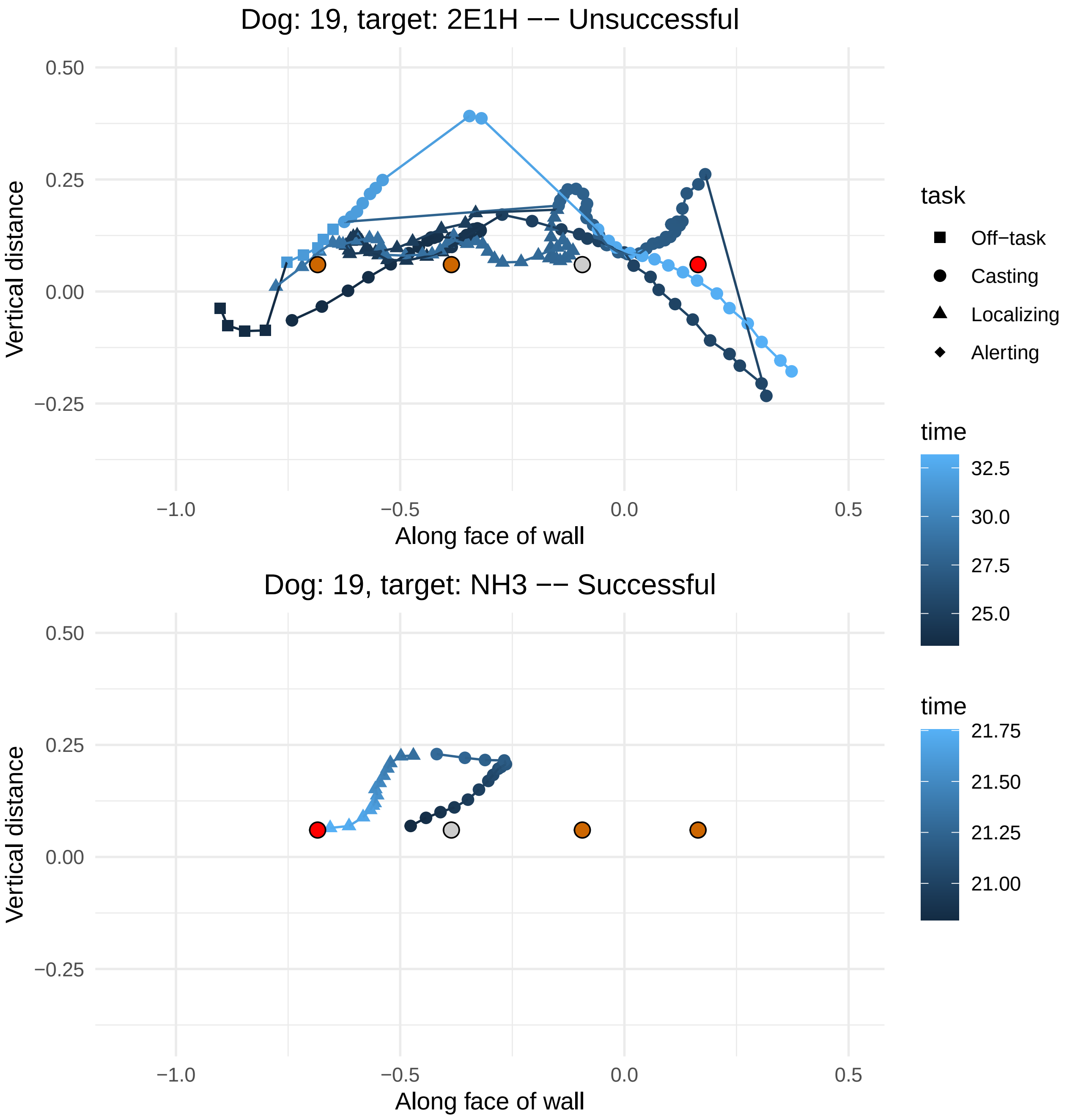


SI Figure 20: Kinematic tracks of dog 19 for both target chemicals.

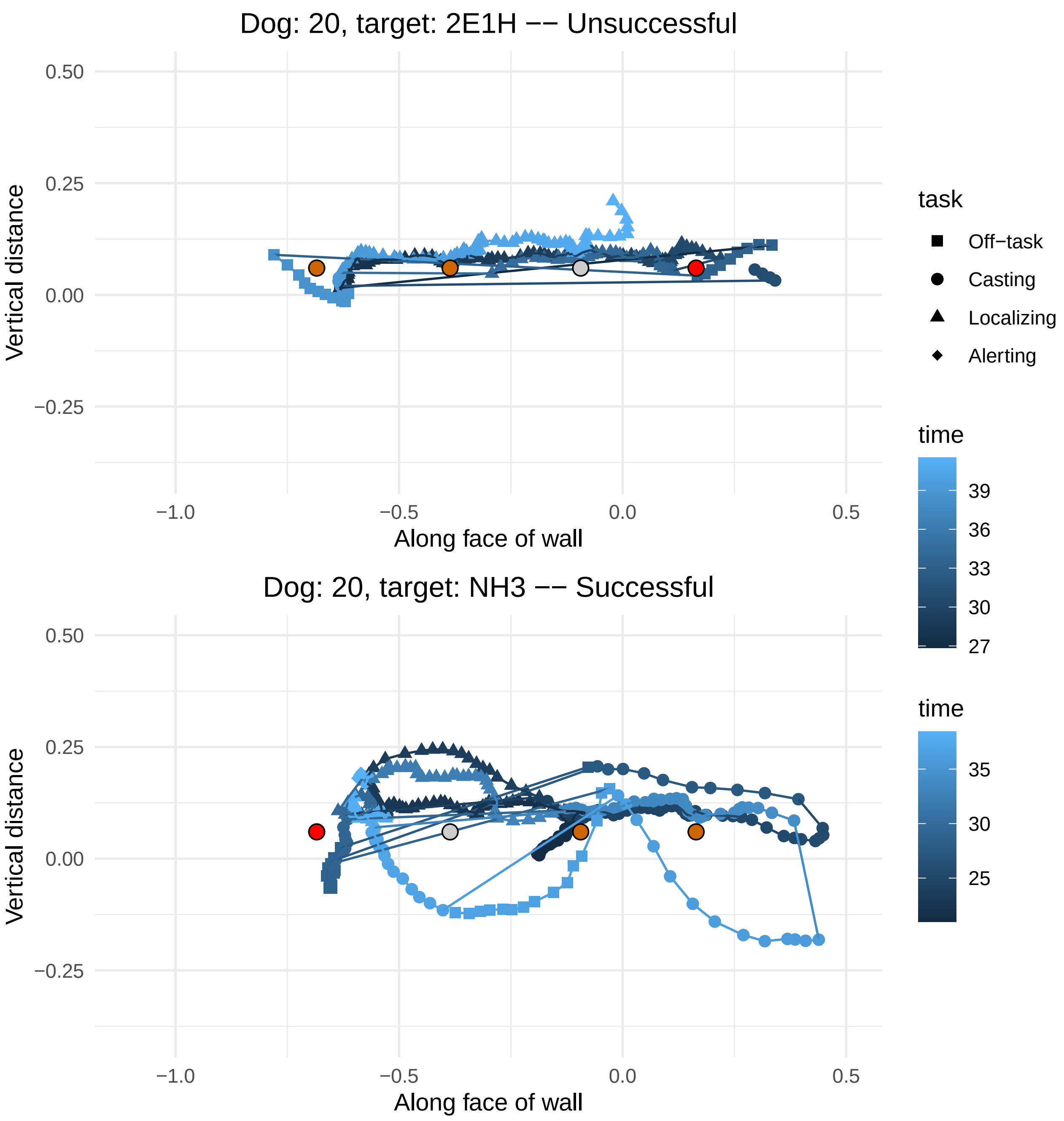


SI Figure 21: Kinematic tracks of dog 20 for both target chemicals.

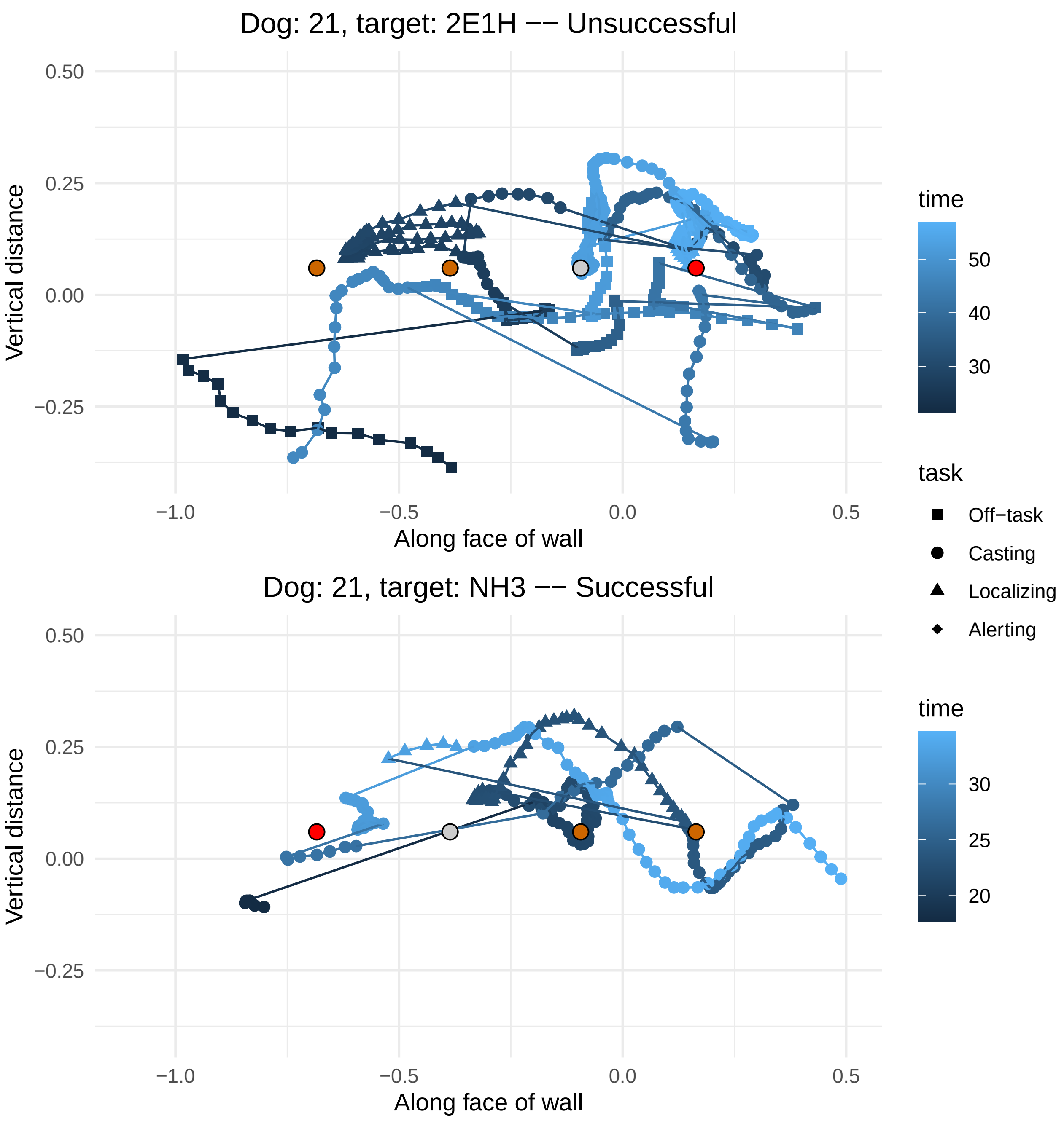


SI Figure 22: Kinematic tracks of dog 21 for both target chemicals.

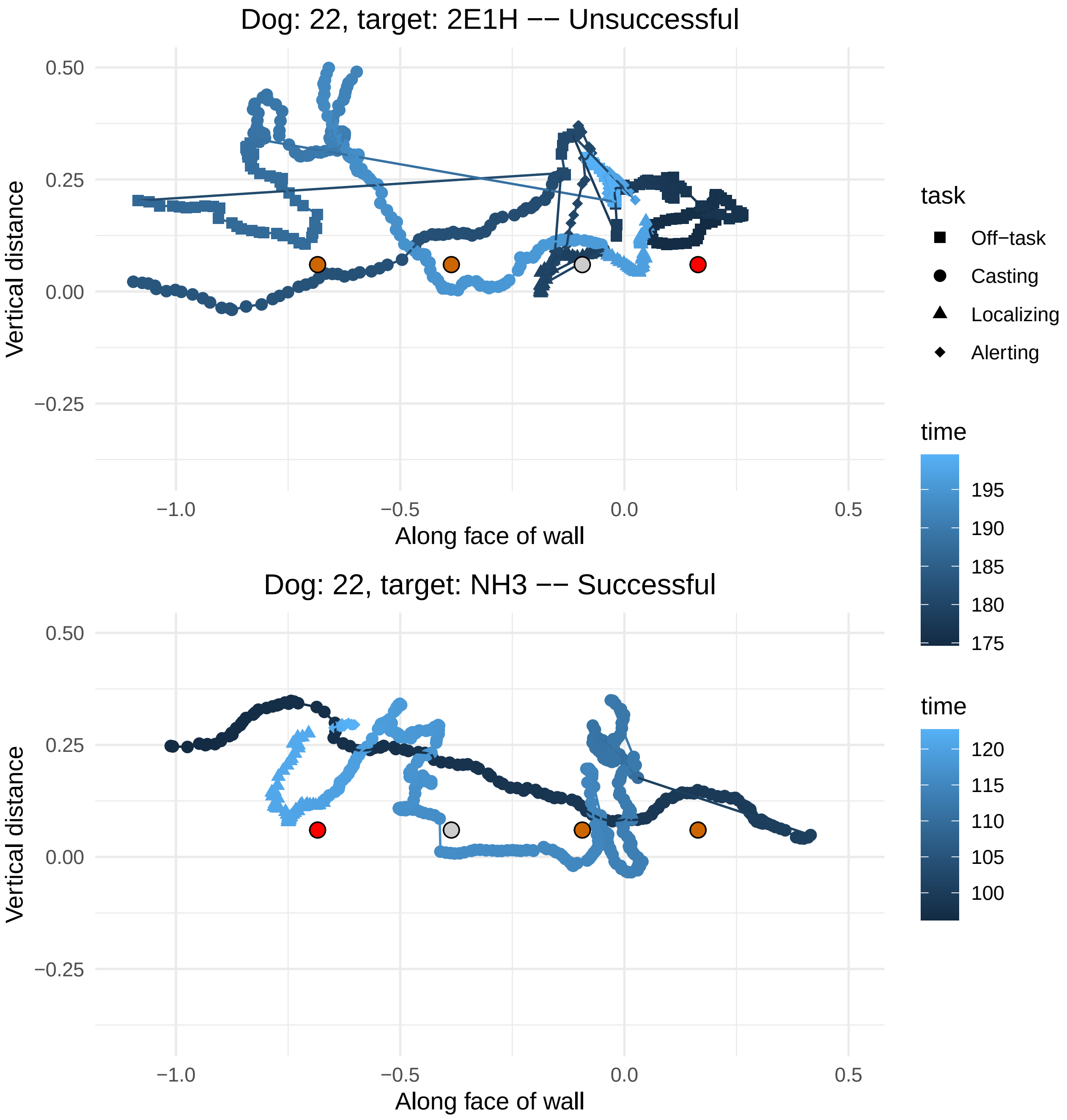


SI Figure 23: Kinematic tracks of dog 22 for both target chemicals.

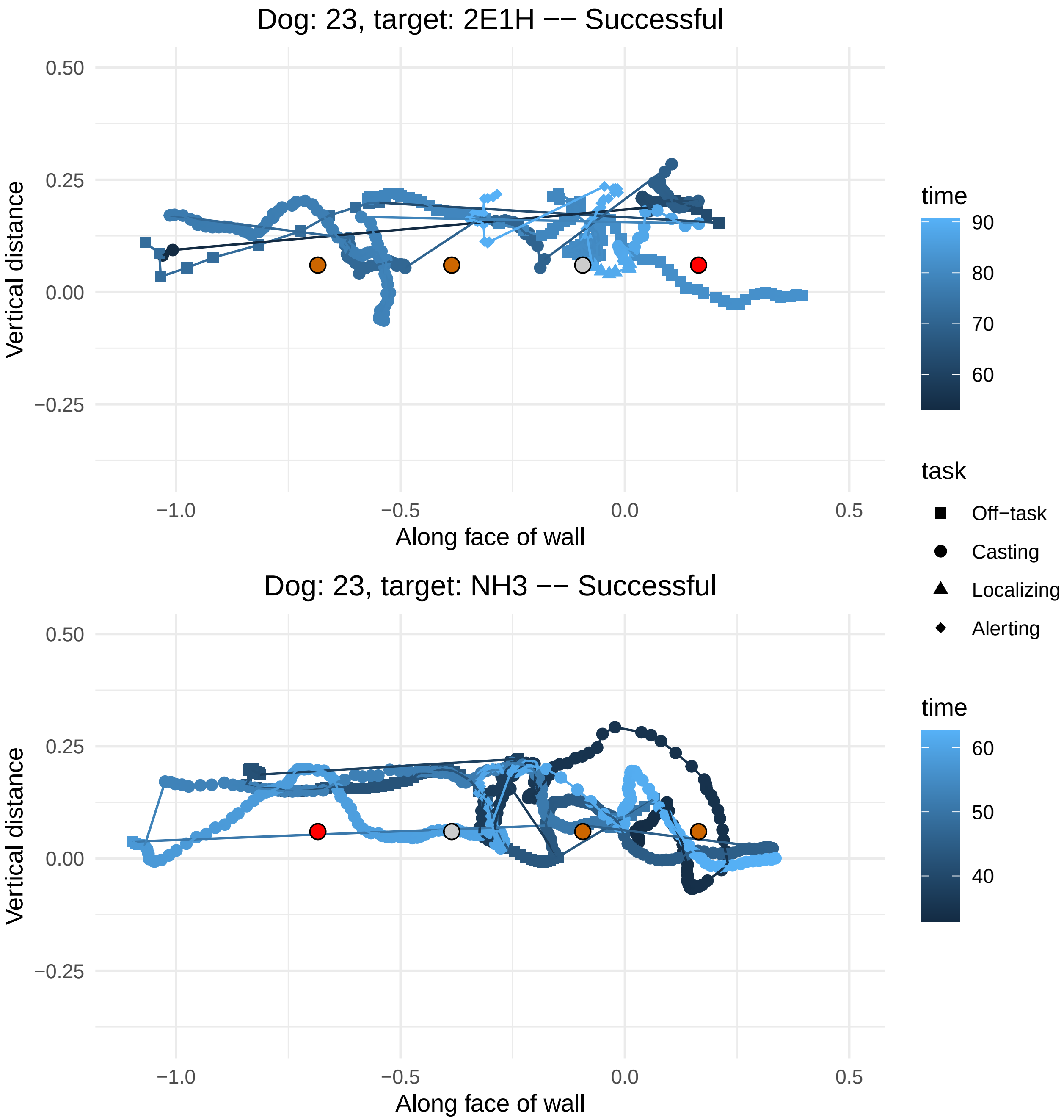


SI Figure 24: Kinematic tracks of dog 23 for both target chemicals.

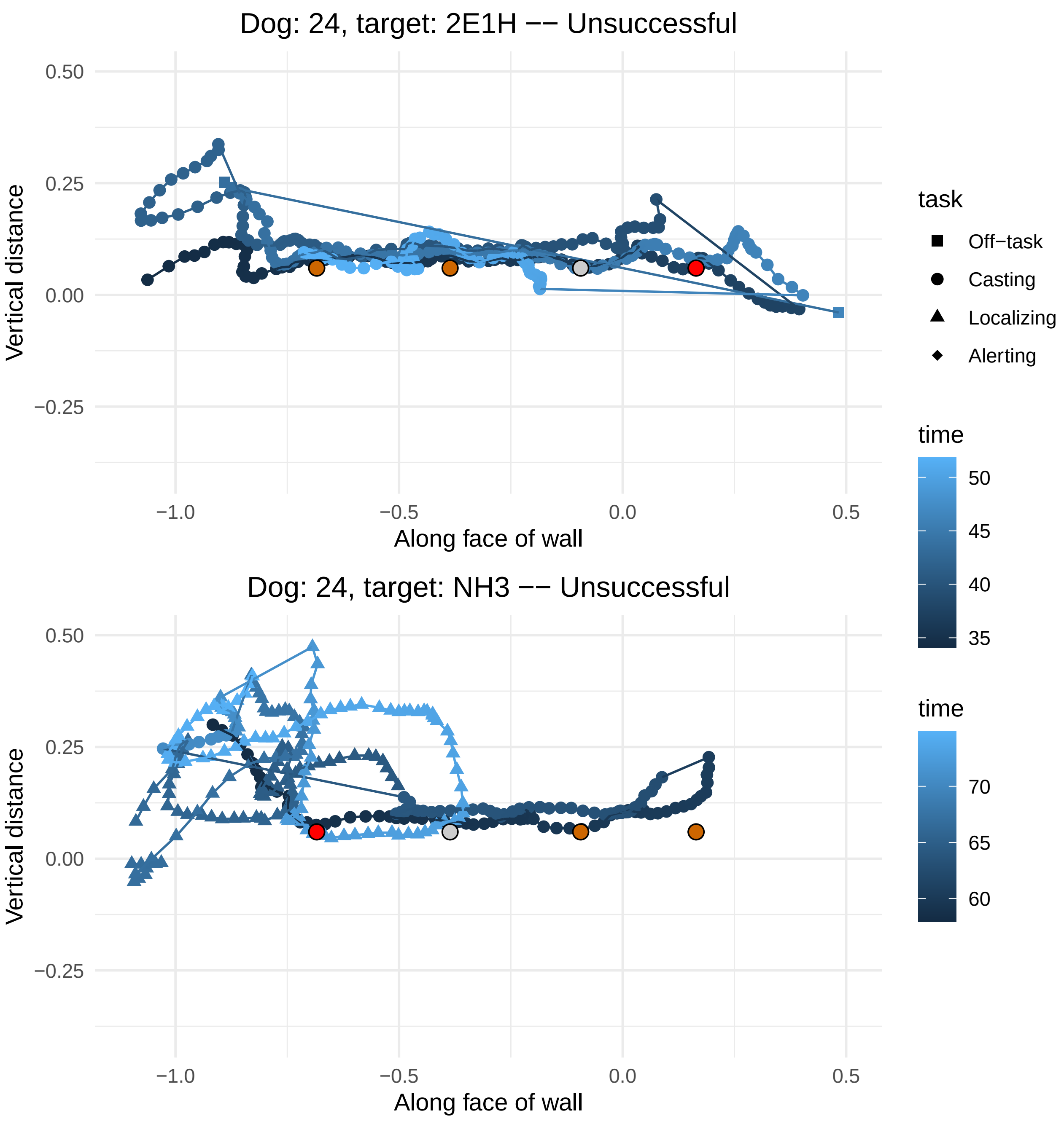


SI Figure 25: Kinematic tracks of dog 24 for both target chemicals.

21. Hartigan, J. A. & Wong, M. A. Algorithm AS 136: A k-means clustering algorithm. *Journal of the Royal Statistical Society. Series C (Applied Statistics)* **28**, 100–108 (1979).

22. Maechler, M., Rousseeuw, P., Struyf, A., Hubert, M. & Hornik, K. *Cluster: Cluster Analysis Basics and Extensions.* vols Version 2.1.4 (2022).

23. Tibshirani, R., Walther, G. & Hastie, T. Estimating the Number of Clusters in a Data Set Via the Gap Statistic. *Journal of the Royal Statistical Society Series B: Statistical Methodology* **63**, 411–423 (2001).
